# Actinium-225 targeted alpha particle therapy for prostate cancer

**DOI:** 10.7150/thno.96403

**Published:** 2024-05-11

**Authors:** Anil P. Bidkar, Luann Zerefa, Surekha Yadav, Henry F. VanBrocklin, Robert R. Flavell

**Affiliations:** 1 Department of Radiology and Biomedical Imaging, University of California San Francisco, CA-94107, USA.; 2 UCSF Helen Diller Family Comprehensive Cancer Center, San Francisco, CA-94107, USA.; 3 Department of Pharmaceutical Chemistry, University of California, San Francisco, CA-94107, USA.

**Keywords:** Actinium-225, targeted alpha therapy, prostate cancer, alpha particle therapy

## Abstract

Targeted alpha particle therapy (TAT) has emerged as a promising strategy for the treatment of prostate cancer (PCa). Actinium-225 (^225^Ac), a potent alpha-emitting radionuclide, may be incorporated into targeting vectors, causing robust and in some cases sustained antitumor responses. The development of radiolabeling techniques involving EDTA, DOTA, DOTPA, and Macropa chelators has laid the groundwork for advancements in this field. At the forefront of clinical trials with ^225^Ac in PCa are PSMA-targeted TAT agents, notably [^225^Ac]Ac-PSMA-617, [^225^Ac]Ac-PSMA-I&T and [^225^Ac]Ac-J591. Ongoing investigations spotlight [^225^Ac]Ac-hu11B6, [^225^Ac]Ac-YS5, and [^225^Ac]Ac-SibuDAB, targeting hK2, CD46, and PSMA, respectively. Despite these efforts, hurdles in ^225^Ac production, daughter redistribution, and a lack of suitable imaging techniques hinder the development of TAT. To address these challenges and additional advantages, researchers are exploring alpha-emitting isotopes including ^227^Th, ^223^Ra, ^211^At, ^213^Bi, ^212^Pb or ^149^Tb, providing viable alternatives for TAT.

## Introduction

Prostate cancer (PCa) is the most common non-cutaneous malignancy among men, and a significant threat to public health. PCa is projected to be diagnosed in approximately 299,010 men within the United States in 2024, accounting for approximately 29% of all malignancies 1. The lethality of prostate cancer stems from its capacity to metastasize, leading to significant health challenges and loss of life. Despite the advances in diagnostic techniques, including prostate-specific antigen (PSA) screening, a substantial proportion of patients still present with aggressive forms of the disease, necessitating better and alternative therapeutic strategies. Effective management of prostate cancer involves a diverse array of treatment modalities tailored to specific characteristics and disease stage 2. For localized or early-stage prostate cancer, treatment options may include active surveillance, cryosurgery, brachytherapy or external beam radiotherapy, radical prostatectomy (surgical removal of the prostate gland), hormone therapy, or other focal therapies aimed at targeting specific areas of the prostate 2,3. However, in metastatic disease, curative options become limited, and the challenges of treatment increase. Conventional therapies, such as surgery, radiation, and hormone therapy, may effectively control localized disease or delay progression, but they often are not cancer-specific and come with significant side effects, which may not be suitable for all patients.

The urgency for more precise and effective treatment options has driven the emergence of targeted therapies in oncology. These therapies aim to exploit specific molecular features of cancer cells to deliver highly potent and selective treatments. Prostate-Specific Membrane Antigen (PSMA), a type II transmembrane glycoprotein has revolutionized diagnostic, prognostic, and therapeutic approaches 4. With its elevated and selective overexpression in prostate cancer cells, PSMA stands as an important target for precision medicine strategies. The ectodomain of PSMA exhibits glutamate carboxypeptidase enzymatic activity, mediating the cleavage of N-acetylated-L-aspartyl-L-glutamate (NAAG), thereby potentially influencing tumor microenvironment modulation 5. Its overexpression is attributed to androgen receptor signaling and other molecular mechanisms and substantiates its potential as a therapeutic target. PSMA-targeted imaging using radiopharmaceuticals has revolutionized prostate cancer detection 6. PSMA targeted PET imaging is now standard of care in prostate cancer.

More recently, PSMA targeted radiopharmaceutical therapy has emerged as a powerful therapeutic modality in advanced metastatic castration resistant prostate cancer. The phase II TheraP study compared [^177^Lu]Lu-PSMA-617 with cabazitaxel in metastatic castration-resistant prostate cancer 7. [^177^Lu]Lu-PSMA-617 showed higher PSA responses (66% vs 37%) and fewer severe adverse events (33% vs 53%) compared to cabazitaxel 8. The findings suggest [^177^Lu]Lu-PSMA-617 as a promising and well-tolerated alternative to cabazitaxel for this patient population. Subsequently, the VISION trial marked a substantial advancement in the ongoing battle against mCRPC. This pivotal international effort was a randomized, open-label phase III trial that successfully recruited 831 men diagnosed with PSMA-positive mCRPC 9. The participants were divided into two treatment groups: one receiving [^177^Lu]Lu-PSMA-617 in conjunction with the standard of care (n=551), and the other undergoing the standard of care alone (n=280). The groundbreaking results of the trial revealed a significant improvement in progression-free and overall survival for those treated with [^177^Lu]Lu-PSMA-617 alongside standard of care compared to those receiving standard of care alone.

**Figure 1:**
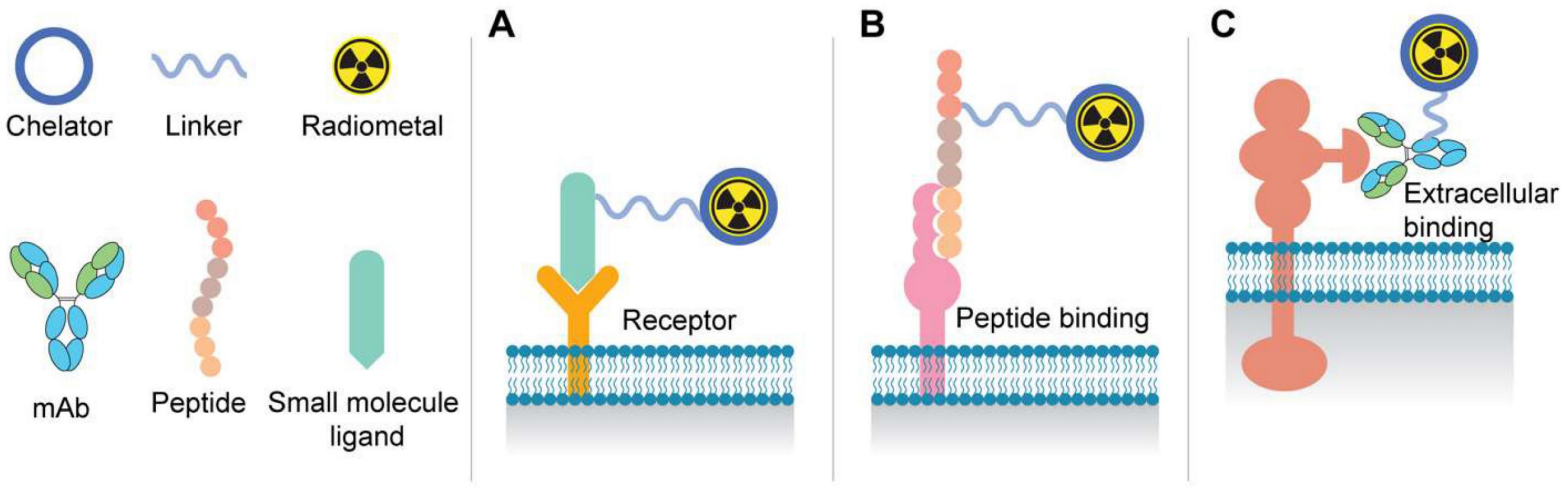
Schematic illustration of radioligand therapy molecules: **A**. Binding of small molecule PSMA ligands to receptor active site on the cell surface. **B**. Representation of the use of peptide-based targeting agents. **C**. Tumor-specific binding of antibodies to the extracellular domain of surface proteins.

**Figure 1** depicts the most common components of the various radioligand modalities including small molecules, peptides, and antibody-based agents for radiotheranostics. Positron Emission Tomography (PET) radiotracers, such as [^68^Ga]Ga-PSMA-11 and [^18^F]-DCFPyL, exhibit high specificity and sensitivity in identifying metastatic lesions and guiding treatment decisions, thereby circumventing the limitations of conventional imaging techniques 10. Recently, the FDA has approved [^177^Lu]Lu-PSMA-617 (Pluvicto) for patients with advanced metastatic castration-resistant prostate cancer (mCRPC) previously treated with chemotherapy and anti-androgen therapy 11. However, the TheraP study, as well as the phase III VISION study, further indicated that 17% and 30% of patients had inherent resistance to the [^177^Lu]Lu-PSMA-617, respectively 12.

**Figure 2:**
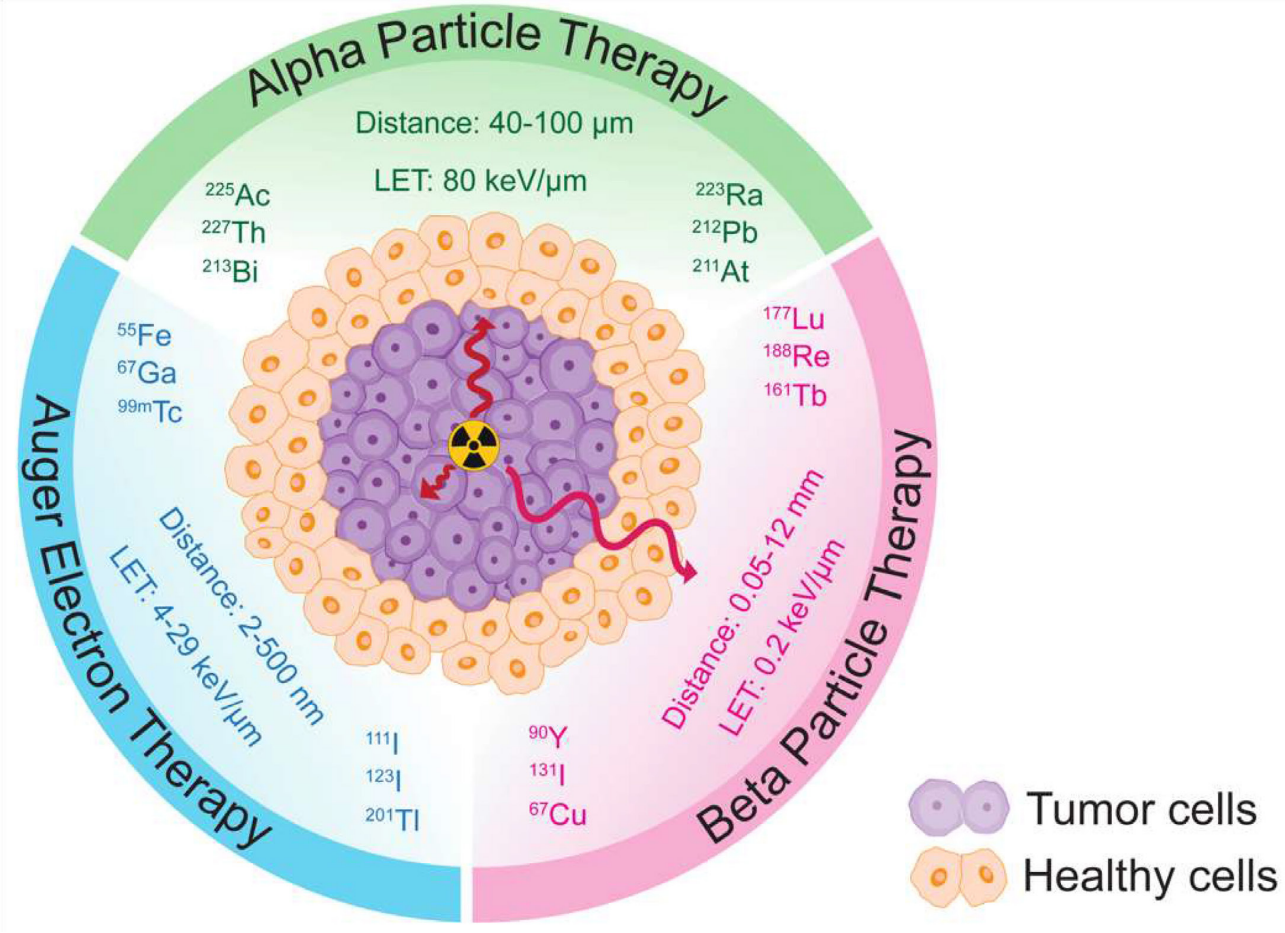
Schematic comparison of the distance traveled and Linear Energy Transfers (LETs) of α, β particles, and Auger electrons in tumor and healthy tissues.

Targeted alpha therapy (TAT) harnesses the unique properties of alpha particle radiation for cancer treatment. Alpha particles are high-energy (LET= 80 keV/µm), highly charged particles that possess a short range (40-100 µm), making them ideal for localized and precise targeting of cancer cells (**Figure 2**) 13. Unlike beta particles (0.2 keV/µm, 0.05-12 mm), alpha particles deposit a significant amount of energy over a very short distance, resulting in potent and localized damage to tumor cells. This characteristic allows for targeted delivery and reduced cross-fire effect to surrounding healthy tissues.

**Figure 3:**
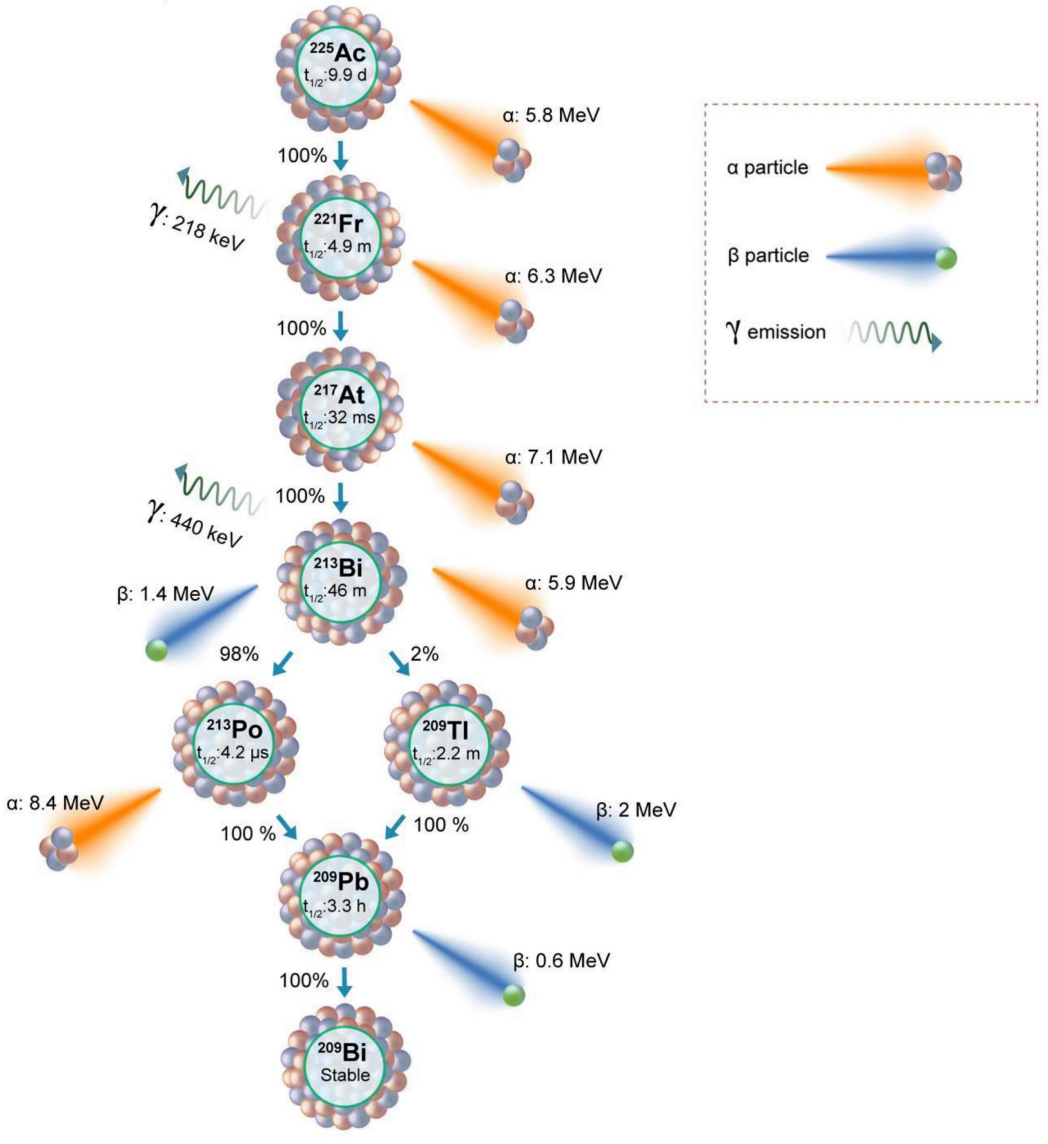
Decay scheme of ^225^Ac showing daughter isotopes, alpha, and beta particle emissions along with the energies.

Actinium-225 (^225^Ac) is a promising alpha-emitting radionuclide for prostate cancer targeted radiotherapy. ^225^Ac has a half-life of 9.92 days, during which it decays through a series of short-lived alpha and beta-emitting daughters (**Figure 3**). This decay chain results in the release of four net alpha particles per decay event, maximizing the therapeutic potential of ^225^Ac. In this review paper, we aim to provide a comprehensive understanding of Actinium-225-based targeted alpha therapy for prostate cancer treatment. By exploring the principles of alpha particle radiation and its advantages in cancer therapy, we seek to emphasize the significance of targeted therapies in prostate cancer treatment. Furthermore, we will delve into the properties of ^225^Ac, the radiolabeling techniques utilized for its efficient delivery, and its role in preclinical and clinical studies. Additionally, we will evaluate the efficacy, safety considerations, and challenges associated with ^225^Ac-based TAT for prostate cancer.

## History and Radiolabeling Methods of ^225^Ac

### Actinium-225 production

**Figure 4:**
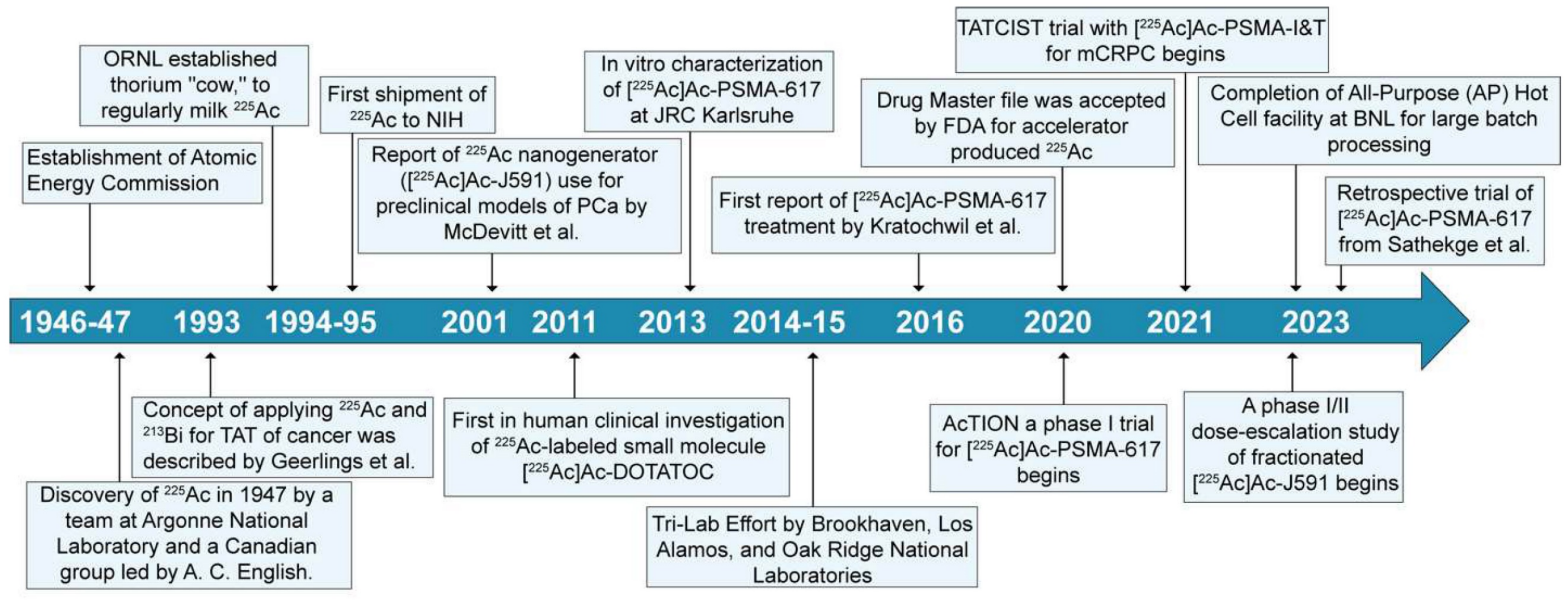
Chronological sequence of significant milestones spanning from the initial identification of ^225^Ac to its current role in prostate cancer.

Actinium-225 was first identified in 1947 by a team at Argonne National Laboratory and a Canadian research group led by A. C. English (**Figure 4**) 14,15. It wasn't until 1993 that the possible use of ^225^Ac and ^213^Bi as radioimmunotherapy (RIT) agents was suggested by Geerlings *et al.* (**Figure 4**) 16. ^225^Ac, characterized by a relatively long half-life, undergoes a decay process involving six short-lived radionuclide daughters, ultimately reaching the near-stable state of ^209^Bi (half-life: approximately 19 quintillion years) 17. The four net alpha particles, two beta emissions, and two gamma emissions generated from ^225^Ac decay render it as an important radioisotope for therapeutic applications (see **Figure 3**) 18. The ^225^Ac decays (alpha particle emission with E_α_ of 5.8 MeV, t_1/2_=9.9 days) to ^221^Fr (E_α_ 6.3 MeV, t_1/2_=4.8 min), and ^217^At (E_α_ 7.1 MeV t_1/2_=33 ms) followed by ^213^Bi (E_α_ 5.8 MeV, 45.6 min). ^213^Bi decays with branched beta decay (branching ratio 98%, E_β_ 1.4 MeV) to alpha emitter ^213^Po, and alpha decay (2%, E_α_ 5.9 MeV) to ^209^Tl. Finally, both ^213^Po (8.4 MeV, t_1/2_= 3.7 µs) and ^209^Tl decay to ^209^Pb (E_β_ 1.4 MeV), followed by stable ^209^Bi 18. Two gamma emissions from ^225^Ac daughters, ^221^Fr (218 keV) and ^213^Bi (440 keV) are utilized for imaging applications. Additionally, the alpha particles have limited penetration (shorter path length, 40-100 µm), making them highly effective in delivering targeted radiation to localized cancer cells while minimizing damage to surrounding healthy tissues to reduce off-target toxicity. Among the daughters, ^213^Bi has gained more attention, and TATs based on ^213^Bi are under preclinical evaluation 19.

The approval of ^223^RaCl_2_ (Xofigo) by the FDA in 2013 for managing prostate cancer bone metastases in castration-resistant prostate cancer patients sparked a resurgence of interest in TATs. Despite the successful use of ^223^RaCl_2_, incorporation of ^223^Ra into targeted agents was not established due to its challenging chelation chemistry 20. However, other α-emitting radionuclides, including ^227^Th, ^225^Ac, ^213^Bi, ^212^Bi, ^212^Pb, and ^211^At, have more suitable chemical properties for attachment to targeting vectors. The use of these radioisotopes requires the formation of a thermodynamically stable and kinetically inert complex with a chelator. Limited knowledge of the Ac^3+^ ion coordination chemistry made it difficult to find optimum chelators for a stable complex. Additionally, with a large ionic radius (112 pm, 1.12 Å) and low charge-to-ionic ratio, Ac^3+^ tends to display weak electrostatic interactions with the chelator 20. The daughter isotopes from ^225^Ac also experience recoil energy due to the conservation of momentum, which leads to a break in the coordination and subsequent release of the daughter isotope from the chelator 21. Therefore, finding a chelator providing sufficient stability has been an active challenge in the field of TAT with ^225^Ac. Additionally, chelator development research was slowed due to limited access to a regular supply of ^225^Ac.

### Chelation chemistry

**Figure 5:**
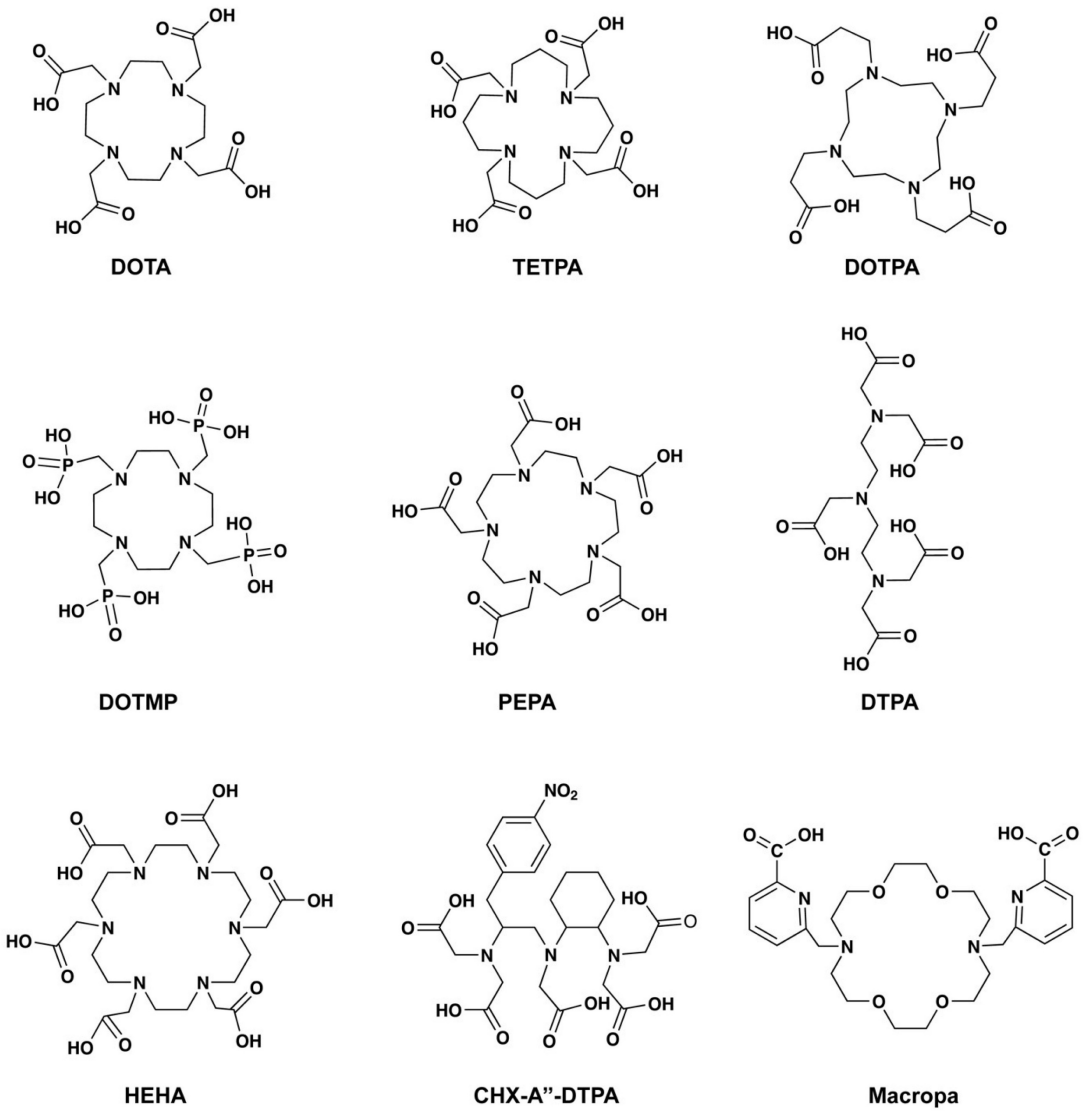
Chemical structures of the chelators used for ^225^Ac radiolabeling.

Early studies in 1999 reported by Davis *et al.* compared the biodistribution of the ^225^Ac-acetate with radiolabeled chelators such as ethylene diamine tetraacetic acid (EDTA), 1, 4, 7, 10, 13- pentaazacyclopentadecane-N, N′, N″, N‴, N⁗-pentaacetic acid (PEPA), and cyclohexyl diethylenetriamine pentaacetic acid (CHX-DTPA) (**Table 1**, **Figure 5**) 22. The radiolabeling of EDTA, PEPA, and CHX-PEPA carried out in 1M NH_4_OH (pH=5) resulted in 80-90% radiochemical yield. CHX-DTPA and PEPA conjugates showed reduced uptake in the liver compared to the ^225^Ac-acetate and EDTA, showing in-vivo stability. In 1999, another study by Deal *et al.* reported the development of 1,4,7,10,13,16-hexaazacyclohexadecane-N,N′, N′′,N′′′,N′′′′,N′′′′′- hexa acetic acid (HEHA) for ^225^Ac complexation 23. The use of the HEHA-mAb antibody conjugate resulted in labeling yield of 60-85% with a specific activity of 200-400 μCi/mg protein, in 0.15 M NH_4_OAc (pH= 4 to 7, 30 min, 37°C) 24. Following this, McDevitt *et al.* studied diethylenetriaminepentaacetic acid (DTPA), 1,4,7,10- tetraazacyclododecane- 1,4,7,10-tetraacetic acid (DOTA), 1,4,8,11- tetraazacyclotetradecane- 1,4,8,11-tetraacetic acid (TETA), 1,4,7,10-tetraazacyclododecane-1,4,7,10-tetrapropionic acid (DOTPA), 1,4,7,10-tetraazacyclododecane-1,4,7,10-tetramethylene-phosphonic acid (DOTMP), and 1,4,8,11-tetraazacyclotetradecane-1,4,8,11-tetrapropionic acid (TETPA) for their radiolabeling and stability characteristics (**Figure 5**). Among the studied chelators, DOTA showed promising radiolabeling (3 M NH_4_OAc, 37°C, 1.0 mL reaction volume) with improved stability in the 25% serum samples (90% intact after 10 days) 25. Significant improvement of radiolabeling yields and stability was seen with 18-membred macrocycle macropa. The monoclonal antibody trastuzumab and a PSMA ligand (RPS-070) were radiolabeled at room temperature in 5 minutes, with 99% retention of ^225^Ac after 7 days 26. Recent studies demonstrate ongoing efforts to use additional macrocyclic chelators including crown, macropid, macrodipa, and py-macrodipa 27.

Since the early 1990s, extraction of ^225^Ac from ^229^Th has assumed a pivotal role in the production of both ^225^Ac and ^213^Bi. Subsequently, clinical and preclinical studies have depended on the ^225^Ac derived through the decay of ^229^Th 19. Globally, there exist three sources of ^229^Th facilitating the generation of ^225^Ac of clinical relevance. These sources are strategically located at the Directorate for Nuclear Safety and Security of the JRC of the European Commission in Karlsruhe, Germany, the Oak Ridge National Laboratory (ORNL) in the United States, and the Institute of Physics and Power Engineering (IPPE) in Obninsk, Russia. The origins of the ^229^Th sources trace back to aged ^233^U, initially synthesized for weaponry applications via neutron irradiation of natural ^232^Th.

The escalating demand for ^225^Ac has drawn the attention of researchers, medical practitioners, and the global patient population, all awaiting the provision of this crucial radionuclide. Their focus revolves around its subsequent application in tailoring patient-specific therapeutic regimens through radiolabeling methodologies. There has been a global response to produce ^225^Ac to meet the potential future demands fueled by TAT. A comprehensive update on ^225^Ac production is provided later in this review.

## Actinium-225 based TAT Agents for Prostate Cancer Tumor Models

### Preclinical studies evaluating small molecule TAT agents

PSMA is a prime target for radioligand therapy (RLT). Radionuclides like ^177^Lu and ^225^Ac, conjugated with PSMA-targeting ligands, enable selective irradiation of tumor cells while minimizing damage to healthy tissues. Since the alpha particle possesses high LET and shorter path length, TAT with ^225^Ac may demonstrate enhanced therapeutic efficacy. A range of α-emitting radionuclides, namely ^149^Tb, ^211^At, ^212^Pb/^212^Bi, ^225^Ac, and ^227^Th, are currently undergoing rigorous assessment for PSMA-targeted alpha particle therapy. PSMA is also a unique target in that it accommodates bulky groups conjugated to the molecule that engages the protein binding site. This property has been accessed to develop a variety imaging and therapeutic PSMA targeted agents.

The utilization of mouse models in PCa research has yielded valuable insights into cancer mechanisms, therapeutic strategies, and treatment advancements. However, the choice of model often limits its clinical applicability. Subcutaneous xenograft models facilitate convenient tracking of tumors and responses but cannot mimic metastatic progression. On the other hand, orthotopic and systemic models offer a clinically relevant context despite being more challenging to establish and monitor. In the context of PSMA-targeted alpha therapy within a murine metastatic prostate cancer model, the timing of treatment emerged as a pivotal factor influencing therapeutic outcomes 28. Stuparu *et al.* showed that administering [^225^Ac]Ac-PSMA-617 in the early stage of C4-2 metastatic tumors holds promise for treating disease with significant survival benefit 28. The use of systemic models is preferable due to their ability to replicate clinical conditions better and provide a precise assessment of treatment responses and interactions.

Due to its relatively low molecular weight, PSMA is readily eliminated through the renal excretion pathway. While a high clearance rate is advantageous for imaging radiotracers, it may inadvertently lead to diminished radiation dose deposition within tumors. To enhance the radiation dose targeted at tumors, modifications are being explored in PSMA ligands to prolong their circulation and tumor retention times. A study by Meyer *et al.* demonstrated that incorporating an extended linker with supplementary naphthyl groups enhances ligand binding to albumin, thereby extending circulating half-life 29. Using a similar strategy, Busslinger *et al.* developed ibuprofen containing ligand [^225^Ac]Ac-SibuDAB, which showed similar radiolabeling and hematological effect as [^225^Ac]Ac-PSMA-617, whereas the tumor uptake of the [^225^Ac]Ac-SibuDAB doubled at 48 h as compared to [^225^Ac]Ac-PSMA-617 (64 ± 11% IA/g vs. 31 ± 3% IA/g), resulting in improved therapeutic response and overall survival in animal models 30. Another study with macropa chelator conjugated to albumin binding unit (4-(p-iodophenyl)butyrate) and one or two PSMA ligands also showed that the uptake of an albumin-bound [^225^Ac]Ac-PSMA molecule was four times higher than the non-albumin binding counterpart 31. A similar albumin-binding PSMA-targeting ligand, RPS-074, labeled with ^225^Ac, achieved complete remission in 6 out of 7 LNCaP xenografts with a single administration of 148 kBq 32.

### Antibody-based Radioimmunotherapy agents for Preclinical Tumors of PCa

Radioimmunotherapy (RIT) combines the precision of monoclonal antibodies with the robust cell-destructive potential of radiation therapy. Large molecules like monoclonal antibodies with higher molecular weights typically exhibit extended circulation within the bloodstream and prolonged bodily retention. By incorporating ^225^Ac, characterized by its long half-life, onto RIT agent, the therapy gains the added advantage of sustained radiation emission over an extended timeframe. The selection of the chelator for radioimmunotherapy becomes essential, as it plays a crucial role in securely binding and holding the radioactive isotope to the monoclonal antibody. Seminal work by McDevitt *et al.* demonstrated that the internalizing anti-PSMA antibody [^225^Ac]Ac-J591 acts as a nanogenerator, producing a series of α particles for maximum radiation dose 33. In vitro experiments conducted in this study revealed that internally localized [^225^Ac]Ac-J591 in LNCaP cells retained the daughter isotopes along with their corresponding α emissions. However, it was noted that daughters produced by [^225^Ac]Ac-J591 bound to the cell surface were likely to be transferred to other sites. Additionally, [^225^Ac]Ac-J591 exhibited a significant anti-tumor response in LNCaP tumor models, leading to improved survival rates and a substantial decrease in PSA levels. This agent is now in clinical trials as outlined below.

The significance of tumor PSMA expression is underscored by its far-reaching implications for both diagnostic and therapeutic strategies. The absence or downregulation of PSMA directly impacts the efficacy of PSMA-targeted approaches 34. Consequently, a pressing need has arisen to explore alternative targets that can facilitate the development of efficacious theranostic strategies. In response to this, notable progress has been achieved through the development of antibodies that target prostate cancer regardless of their PSMA status. One instance of this approach is the development of a full-length IgG antibody targeting CD46, as demonstrated by Su et al 35. CD46 protein was found to be overexpressed on PCa tissues, and the anti-CD46 antibody effectively binds to PCa irrespective of PSMA expression 35. For TAT of prostate cancer, the CD46 targeting [^225^Ac]Ac-DOTA-YS5 antibody, labeled with ^225^Ac, was harnessed 36. The therapeutic agent proved efficacious in reducing tumor volume, exhibiting efficacy against both cell-derived (22Rv1 and DU145) and patient-derived adenocarcinoma (LTL-484) and neuroendocrine prostate cancer (LTL-545) models. Bidkar *et al.* also showed that fractionation of [^225^Ac]Ac-DOTA-YS5 into multiple smaller doses of 0.125 µCi results in a better therapeutic response with lesser toxicity. In addition to this, a surrogate imaging probe [^89^Zr]Zr-DFO-YS5 has been developed to image CD46-positive tumors 37. Recently, Bobba *et al.* utilized a PEGylated Macropa chelator (18-membered macrocyclic) conjugated to an anti-CD46 antibody, demonstrating highly efficient radiolabeling (96% yield) at room temperature within a short timeframe of 5 minutes 38. This approach surpassed the performance of DOTA conjugates and exhibited superior antitumor efficacy. The CD46 target has proven effective for the tumor-targeted delivery of radiation. In a related study, Li *et al.* demonstrated the therapeutic efficacy of a similar alpha emitting isotope, ^212^Pb, conjugated to an anti-CD46 antibody ([^212^Pb]Pb-TCMC-YS5) 39. This novel construct showed promising therapeutic effects with a single dose in both subcutaneous mCRPC cell line-derived and patient-derived xenograft models.

The hK2 is a serine protease expressed in prostate tissue that has an 80% amino acid sequence similar to PSA. The antibody hu11B6 targets hK2, an epitope expressed exclusively on prostate tissue and cancer in vivo. Additionally, it is regulated by the androgen receptor (AR) activity. McDevitt et al*.* prepared the [^225^Ac]Ac-hu11B6 (with DOTA chelator), which delivers radiation specifically to cancer cells, thereby initiating a DNA damage response (DDR) 40. Subsequently, the DDR causes upregulation of the hK2, resulting in feed-forward alpha particle radiotherapy. The [^225^Ac]Ac-hu11B6 therapy took advantage of the unique phenomenon of increasing expression of AR and hK2 from alpha particle-induced DNA damage in the LNCaP-AR tumor model. The hK2 targeting antibody has also been utilized to make an imaging agent [^89^Zr]Zr-hu11B6 to use as a surrogate reporter for [^225^Ac]Ac-hu11B6 and beta particle therapy agent [^177^Lu]Lu-m11B6 40,41. The authors also studied the expression of AR-governed biomarkers in treatment-naïve tumors vs. relapsing tumors. Genes under the control of androgen receptor (AR), namely FOLH1, KLK3, PMEPA1, and SPOCK1, displayed a down-regulated pattern, whereas a subset of androgen-repressed genes, including PMP22, CAMK2N1, and UGT2B17, demonstrated an up-regulated expression 42. Unlike the loss of PSMA in treatment-emergent neuroendocrine/small cell prostate cancer, hK2 (KLK2) expression was intact in the tumors of mice from [^225^Ac]Ac-hu11B6 treatment. The hK2 targeting alpha particle RIT agent ([^225^Ac]Ac-DOTA-h11B6) is currently under phase I clinical trial 43.

Since the expression of hK2 and PSA is regulated due to AR activity, it has led to the utilization of PSA as a target for RIT using an antibody. Veach *et al.* compared the therapeutic efficiency of two types of antibodies targeting PSA: high linear energy transfer (LET) [^225^Ac]Ac-hu5A10 with an LET of approximately 100 keV/µm, and low LET [^90^Y]Y-hu5A10 with a LET of about 0.2 keV/µm 44. This comparison conducted in LNCaP-AR xenografts showed that both the high LET and low LET antibodies resulted in improved median survival when compared to saline control (32 days). Notably, the high LET antibody achieved a significantly higher median survival (188 days) compared to the low LET antibody, showcasing the superiority of [^225^Ac]Ac-hu5A10. Moreover, the study found that a substantial percentage of animals exhibited a complete response to the high LET [^225^Ac]Ac-hu5A10 (38.9%), whereas the low LET [^90^Y]Y-hu5A10 achieved a complete response in only 11.1% of animals.

In the context of addressing solid tumors, an additional emerging therapeutic strategy involves inhibiting tumor angiogenesis. An intriguing approach revolves around vascular endothelial (VE) cadherin, a molecule exclusive to vascular endothelial cells that are consistently expressed throughout the vascular network. VE plays a pivotal role in the establishment of adherens junctions between neighboring endothelial cells. A monoclonal antibody, E4G10, has been developed to specifically bind to an epitope exposed solely to the monomeric, unengaged form of VE-cadherin 45. This epitope becomes concealed upon transdimerization, leading to the formation of inter-cellular junctions. This distinctive feature permits the precise targeting of endothelial cells within nascent tumor vasculature, presenting a selective strategy to hinder tumor growth. Using this approach, Jaggi et al*.* designed [^225^Ac]Ac-E4G10 antibody targeting neovasculature, which showed a significant reduction of LNCaP subcutaneous tumor xenografts.

While RIT agents effectively target tumors, a notable concern is the prolonged circulation time, particularly in the context of ^225^Ac delivery. Optimizing the circulation duration is crucial, and in certain cases, a faster clearance would be beneficial for improved therapeutic outcomes.

## Clinical Studies Testing ^225^Ac TAT Agents in Prostate Cancer Patients

### [^225^Ac]Ac-PSMA-617

In a first description of the use of [^225^Ac]Ac-PSMA-617 for patients, Kratochwil *et al.* presented two cases of mCRPC patients who had previously received either conventional chemotherapy or [^177^Lu]Lu-PSMA-617 treatment 46. Notably, the first patient exhibited the disappearance of PSMA-positive lesions on PSMA PET/CT and a remarkable decline in PSA levels from over 3,000 ng/mL to 0.26 ng/mL within two months of treatment of [^225^Ac]Ac-PSMA-617. Subsequent consolidation therapy with [^225^Ac]Ac-PSMA-617 led to further PSA reduction and a complete PSMA PET/CT imaging response. The second patient, having exhausted conventional treatments and [^177^Lu]Lu-PSMA-617 regimens, entered the study with elevated PSA levels at 419 ng/mL. Treatment with [^225^Ac]Ac-PSMA-617 across three cycles led to a full response in this patient, with PSA levels reaching below 0.1 ng/mL. These findings underscored the potential of [^225^Ac]Ac-PSMA-617 in mCRPC or [^177^Lu]Lu-PSMA-617-resistant patients. More recently, Lawal *et al.* published a study involving 106 patients with skeletal metastasis, revealing that 80% of them exhibited a favorable antitumor response to [^225^Ac]Ac-PSMA-617 47.

Though response can be favorable in patients treated with [^225^Ac]Ac-PSMA-617, toxicities including xerostomia present a significant challenge. Despite favorable PSA responses, severe xerostomia resulted in discontinuation of [^225^Ac]Ac-PSMA-617 therapy for 10% (4/40) 48 and 23% (6/26) 49 patients, reported independently. Another study reported the grade I-II xerostomia for patients treated with [^225^Ac]Ac-PSMA-617 50. Thus, a decrease of PSA in the first few cycles could be followed by a de-escalation of [^225^Ac]Ac-PSMA-617 dose to manage the xerostomia, simultaneously inhibiting tumor growth. The small molecule PSMA ligands show uptake in salivary glands leading to radiation-induced xerostomia. Various strategies are being employed to reduce the uptake of salivary gland hypofunction after RLT or other radiation therapies. Among these options are the external cooling of the glands 51 and the potential use of botulinum toxin A injections to reduce PSMA-targeting compound uptake by lowering salivary gland metabolism 52. Another option involves investigating polyglutamate as a PSMA-binding competitor, with promising initial clinical outcomes 53. Restoring salivary gland function is a third approach, with ongoing investigations into gene transfer of the aquaporin-1 gene 54. Sour stimulation and sialendoscopy with dilatation, saline irrigation, and steroid injections have also been explored, demonstrating beneficial effects on radiation-induced inflammation and quality of life 55. However, even with sialendoscopic support following multiple cycles of TAT, salivary gland function remained diminished, and xerostomia persisted. In addition to this, a combination of low-activity [^225^Ac]Ac-PSMA-617 with full-activity doses [^177^Lu]Lu-PSMA-617 shows improved efficacy and good tolerability in heavily pre-treated mCRPC patients. The approach appeared to enhance treatment response and reduce the xerostomia severity, suggesting that 'tandem therapy' could be an effective strategy 56. Furthermore, the replacement of Glu from the PSMA ligand (Lys-urea-Glu) with Asp or Aad (L-2-aminoadipic acid) has led to the development of a new class of radiotracer probe or therapy agent aimed at reducing salivary gland or kidney uptake. Using this strategy, Kuo *et al.* designed the albumin-binding PSMA targeting agent [^177^Lu]Lu-HTK03149 (Glu replaced with Aad), resulting in significantly higher tumor uptake (145% higher absorbed dose) than [^177^Lu]Lu-PSMA-617, along with lower uptake in salivary glands (0.23 vs 1.78 %IA/g) and kidneys (79.7 vs 7.67%IA/g) at 1h post-injection 57.

Presently, [^225^Ac]Ac-PSMA-617 treatment is often being used as a final resort for mCRPC, after extensive pretreatment involving ADT, chemotherapy, and radioligand therapy with ^177^Lu-PSMA-617. This heavy pretreatment contributes to tumor resistance while also rendering patients more susceptible to adverse effects and toxicities. Chemotherapy-naïve patients with advanced metastatic prostate carcinoma respond to [^225^Ac]Ac-PSMA-617 effectively. The treatment-naïve patients displayed a decline of over 90% in their serum PSA levels after undergoing three or four treatment cycles 58. Conversely, patients whose disease had progressed after receiving two or more previous treatments experienced a less favorable decline in PSA levels following the [^225^Ac]Ac-PSMA-617 treatment. The de-escalation from a fixed activity dose of 8 MBq for the first cycle to 7 MBq to 4 MBq was achieved for the next cycles with positive responses and reduced xerostomia. Using this regimen, a >50% PSA decrease was seen in 91% of patients 50. Molecular changes in the tumor cells including the mutations in the DNA damage repair genes, are considered the factors associated with the resistance to TAT 59. A recent multicenter study conducted by Sathekge *et al.* investigated the treatment outcomes and adverse effects of [^225^Ac]Ac-PSMA-617 in mCRPC patients who had undergone with one or more previous lines of treatment 60. The study revealed that 70% of patients showed any decline in PSA, with 57% of patients showing a decline of 50% or more. The most common side effect observed was xerostomia, followed by bone marrow toxicity 60.

Owing to its longer path, beta particles cross the tumor-affected area and affect the surrounding healthy cells (sometimes termed the crossfire effect), potentially resulting in toxicity. [^177^Lu]Lu-PSMA-617 delivery to bone metastasis may result in significant toxicity to bone marrow leading to hematological complexities. Conversely, alpha particles from [^225^Ac]Ac-PSMA-617 with a shorter range and high LET are considered more useful for micro-metastatic tumors 61. Therefore, [^225^Ac]Ac-PSMA-617 has been an effective treatment for brain metastasis of mCRPC 62.

### J951 for PSMA Targeted Theranostics

In addition to the developments of the small molecule PSMA targeting ligands, various antibodies targeting PSMA have been studied, which mitigate salivary gland toxicity 63-65. Among these, 7E11, a PSMA intracellular domain binding, and J951, an extracellular domain binding antibody, have been extensively tested for clinical applications (**Table 2**) 63. Since then, [^89^Zr]Zr-huJ591 has been shown to localize and detect metastatic lesions in prostate cancer patients, while the β or α particle emitter-loaded antibodies are also being tested clinically 66. A single dose or fractionated dose of [^177^Lu]Lu-J591 showed efficacy in mCRPC patients, with a decline in PSA levels 67,68. Dose escalation studies (NCT04576871, NCT03276572, and NCT04506567) are being performed for single and fractionated doses of [^225^Ac]Ac-J591 in mCRPC patients 69,70. The phase I dose escalation of the [^225^Ac]Ac-J591 involved 32 patients, dosed at 13.3 to 93.3 KBq/kg 71. The results from this study demonstrated that one patient showed dose-limiting toxicity (grade ≥3 nonhematologic or grade 4 hematological) with 80 KBq/kg dose and hematologic adverse events (grade 3) were in correlation with administered radioactivity. This trial demonstrated safety in administering a single dose of [^225^Ac]Ac-J591 to patients with pretreated progressive mCRPC. The safety profile, dosimetry, and therapeutic potential of the combination of the small molecule [^177^Lu]Lu-PSMA I&T and the [^225^Ac]Ac-J591 are also being studied to utilize the complimentary benefits of two therapies in mCRPC patients 72.

## TAT in the hormone-sensitive PCa setting

Various factors such as significant tumor load, low hemoglobin levels, reduced performance condition, and a history of several past therapies all influence RLT outcome and tolerance 77. Consequently, it is hypothesized that PSMA-RLT may be advantageous for patients in an earlier disease stage. A recent prospective phase I study demonstrated promising results, where ten patients in the early stages of hormone-sensitive prostate cancer underwent two cycles of [^177^Lu]Lu-PSMA-617 RLT 78,79. Ongoing Phase 3 trials, including PSMAfore (NCT04689828), SPLASH (NCT04647526), and ECLIPSE (NCT05204927), are actively evaluating the benefits of [^177^Lu]Lu-PSMA-based RLT compared to transitioning to androgen receptor targeted therapy (ARTT). A recent press release from the SPLASH study demonstrated improved radiographic progression-free survival in mCRPC patients who progressed on ARTT and were then treated with RLT rather than switching to ARTT. While these investigations mostly focus on beta emitters, a corresponding concept for alpha emitters is emerging, while research in this field is still in its early stages. In a study evaluating the tandem utilization of [^177^Lu]Lu-PSMA-617 and [^225^Ac]Ac-PSMA-617 in patients with early-stage hormone-sensitive metastatic prostate cancer, 85% of patients exhibited a PSA response of ≥50% following PSMA-RLT 80. This raises hope that future studies evaluating ^225^Ac based RLT alone or in combination with beta emitters might be applied in early prostate cancer.

## Current and Future Combination Therapy Approaches in PCa Treatment

**Figure 6:**
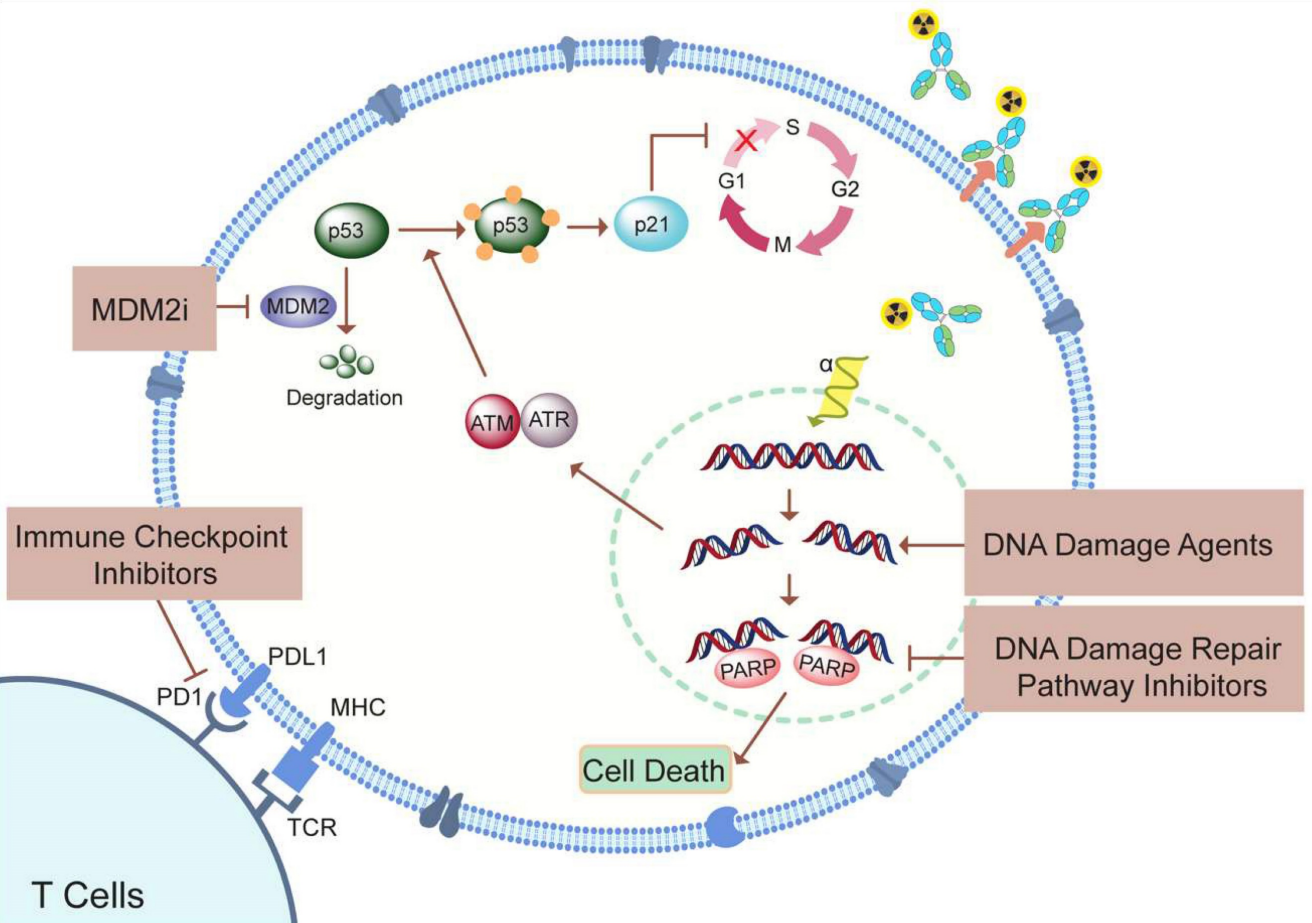
Summary of possible combination therapy approaches to enhance the therapeutic effect of TAT.

Research in the field of targeted radioligand therapy led to clinical approval of agents such as [^177^Lu]Lu-DOTATATE, ^131^I-metaiodobenzylguanidine, Bexxar, and Zevalin, for treating conditions like neuroendocrine tumors (NETs), neuroblastoma, and non-Hodgkin lymphoma. Monotherapy, though the cornerstone, has been challenged by the adaptive plasticity exhibited by prostate cancer cells 81. These cells, adept at reconfiguring their signaling networks, often render single-agent interventions ineffective over time. Given the limitations of monotherapy, various combination therapy strategies are being explored, including enhancing tumor perfusion to enable optimal radiopharmaceutical distribution, upregulating target receptors to augment cellular uptake, combining nuclide therapy with DNA-damaging drugs, inhibiting essential processes like DNA damage repair for radiosensitization, and incorporating immune checkpoint inhibitors (**Figure 6**). One of the approaches is to combine mAb and the small molecule with two different therapeutic radioisotopes targeting the same protein on the cell surface. In this context, the use of mAb ([^225^Ac]Ac-J591) and [^177^Lu]Lu-PSMA-I&T showed improved binding and higher radiation dose delivery compared to a single agent, resulting in a synergistic therapeutic efficiency in xenograft models. Clinical studies are in progress on mCRPC patients to find the maximum tolerated dose for the combination treatment of PSMA targeting [^225^Ac]Ac-J591 and [^177^Lu]Lu-PSMA-I&T (NCT04886986) 70,72. As TAT is known to produce DNA damage as well as activation of immunogenic response, the use of [^225^Ac]Ac-J591 in combination with pembrolizumab (PD-1 inhibitor) is being tested in PCa patients (NCT04946370) 76.

Since the poly (ADP-ribose) polymerase (PARP) enzymes are the first responder to DNA damage, alpha particle therapy may be effectively combined with the PARP inhibitors (PARPi) (**Figure 6**). ^225^Ac, an alpha particle-emitting radionuclide, contributes to this process by inducing highly localized, dense ionization along its path, resulting in complex DNA double-strand breaks. PARPi disrupt DNA repair pathways, particularly in cancers with BRCA mutations, leading to the accumulation of lethal DNA damage. By concurrently inhibiting PARP and exposing cancer cells to the high LET radiation from ^225^Ac, a dual assault on DNA repair mechanisms is orchestrated. The combination of beta particle therapies, with PARPi, like Olaparib, has been a subject of research interest 82,83. A recent phase I study specifically investigated the safety profile of combining Olaparib with ^223^Ra in patients with mCRPC, indicating the growing interest and exploration of this combination in clinical settings 84.

## Challenges and Future Perspectives

### Actinium-225 production

The main source of ^225^Ac has been the stocks of ^229^Th extracted from ^233^U. The milking of ^225^Ac derived from ^229^Th remains insufficient to facilitate the widespread and routine demand. Due to this, alternative approaches to produce ^225^Ac at a large scale are being explored. The accelerator produced ^225^Ac has potential to meet current demand. Firstly, the high-energy proton irradiation (>100 MeV) of ^232^Th produces a large quantity of ^225^Ac, but it concurrently yields other radionuclides through spallation and fission reactions that need to be separated 18,85. Notably, a longer-lived ^227^Ac isotope, with a half-life of 21.8 years, co-produced at a rate of 0.1-0.2% relative to ^225^Ac activity, is the main limitation. In addition to this, efforts are being made to produce ^225^Ac through proton irradiation of ^226^Ra targets. The most promising approach is the use of medium energy proton (20 MeV) accelerators. The irradiation of ^226^Ra targets via the reaction ^226^Ra(p,2n)^225^Ac presents distinct merits compared to the ^232^Th spallation process 86. It can be performed in medium-sized cyclotrons, making it cost-effective without compromising the yield. Proton irradiation of ^226^Ra targets yields pure ^225^Ac without co-production of ^227^Ac. Although short-lived impurities like ^226^Ac (with a half-life of 29 hours) and ^224^Ac (with a half-life of 2.9 hours) are formed, their presence is manageable. Another approach to produce the ^225^Ac is via photonuclear ^226^Ra(ɣ,n)^225^Ra reaction, where generated ^225^Ra decays to ^225^Ac 87. This approach will produce the ^225^Ac without impurities, however in lower yield 87.

In response to the growing worldwide demand and the critical scarcity of this isotope, the United States Department of Energy's Isotope Program (DOE IP) has initiated a comprehensive array of initiatives. These initiatives include strategic facility utilization and judicious allocation of funding, spanning various avenues for the synthesis of ^225^Ac 88. Of particular significance among these ventures is the "Ac-225 Tri-Lab Effort," a synergistic collaboration undertaken by three national laboratories: Oak Ridge National Laboratory (ORNL), Brookhaven National Laboratory (BNL), and Los Alamos National Laboratory (LANL). This collaborative endeavor delves into the feasibility of generating ^225^Ac via accelerator-based methodologies, thereby contributing substantively to addressing the augmented global need. TRIUMF, a Canadian physics laboratory, has also been working towards production of ^225^Ac and other isotopes. Due to the high costs associated with each batch of ^225^Ac production, the current approach for synthesizing ^225^Ac at TRIUMF involves in-house production using the Isotope Separator and Accelerator (ISAC) Facility 19. At TRIUMF, the ^225^Ac is generated through the irradiation of Uranium and Thorium targets with protons, and after additional purification steps, it is employed in TAT studies.

### Quality control of ^225^Ac radiopharmaceuticals

The radiolabeling reactions and percentage purity of ^225^Ac compounds are primarily monitored using radio-thin layer chromatography (radio-TLC). The continuous generation of daughter isotopes (^213^Bi and ^221^Fr) makes it challenging to accurately estimate the radiochemical yield immediately after the run. ^221^Fr, with half-life of 4.9 minutes, reaches secular equilibrium in approximately 1 hr, while ^213^Bi (t_1/2_=45.6 minutes) take hours. Therefore, it is advisable to read the radio-TLCs after attaining secular equilibrium, approximately 4 to 5 hours after the completion of radio-TLC 89. International Atomic Energy Agency (IAEA) also recommends radio-TLC as a quality control test for ^225^Ac labeled peptides 90,91. A study by Yang *et al.* reported that additional quality control tests are needed to use of any radiopharmaceutical with ^225^Ac 91. The [^225^Ac]Ac-crown-αMSH molecule synthesized by Yang et al showed radiolysis due to the high-energy alpha particles, which was not observed on radio-TLC but was evident on radio-high performance liquid chromatography (radio-HPLC). Therefore, the combination of delayed iTLC for radiolabeling, HPLC for the integrity of radiopharmaceuticals, and gamma spectroscopy for the determination of free ^225^Ac are recommended 91. Additionally, retention times of the ^213^Bi and ^221^Fr, determined from fraction collection and gamma counting, could be used to calculate the radiochemical purity using HPLC 91. Furthermore, the use of ROS scavengers like ascorbic acid or reducing the time between purification and dose injection could help reduce radiolysis 91,92. Since the daughters ^213^Bi and ^221^Fr take different times to reach secular equilibrium with ^225^Ac, quantification of ^225^Ac using ^213^Bi and ^221^Fr before equilibrium or in case of ^213^Bi redistribution is challenging. In this regard, to determine the ^225^Ac amount, Seone *et al.* developed a multi-time point gamma counting of ^213^Bi (before secular equilibrium), and single time-point gamma counting of ^213^Bi and ^221^Fr considering no secular equilibrium 93. Along with this, one major quality control concern related to accelerator produced ^225^Ac via spallation of ^232^Th is an impurity of long lived ^227^Ac. Various purification methods are being tested to separate the ^227^Ac from ^225^Ac 18,85,94.

### Recoiling energy, toxicity, and side effects

**Figure 7:**
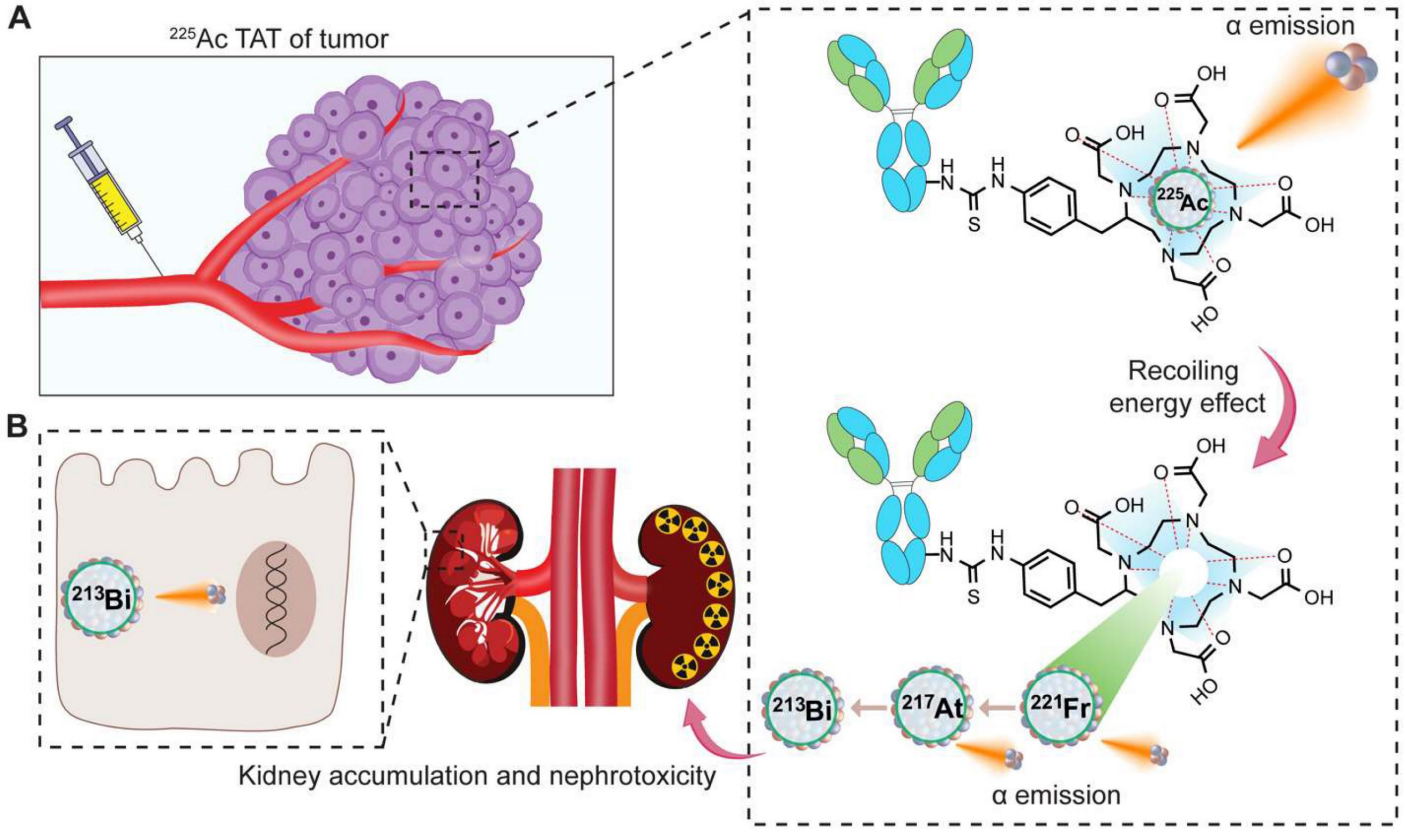
The decay process of ^225^Ac, leading to the emission of alpha particles, induces recoiling energy, subsequently causing the redistribution of daughter isotopes. **A**. Upon injection of the ^225^Ac-based TAT radiopharmaceutical, accumulation occurs in the tumor tissue. The ^225^Ac in circulation or tumor tissues decays, producing alpha particles along with recoiled ^221^Fr and ^213^Bi. **B**. The redistribution of the ^213^Bi contributes to nephrotoxicity.

The toxicity associated with ^225^Ac-based TAT may arise in part from the recoil effect. Although significant progress has been made in developing chelators that create stable and inert complexes with ^225^Ac, the challenge of the recoil effect remains 20. This recoil effect results in the release of the ^221^Fr isotope from the chelate as a result of the alpha emission from ^225^Ac, abiding by the principles of momentum conservation. During the decay process to produce four net alpha particles, the energy released due to the recoil of daughter nuclides exceeds that of more than 10,000 chemical bonds, thereby leading to the separation of daughter nuclides from the chelator (**Figure 7A**) 21. This leads to the distribution of daughter isotopes to non-targeted tissues causing significant toxicity to healthy organs and compromising therapeutic efficacy. Various preclinical and clinical studies report that redistribution of daughter ^213^Bi into the kidney from ^225^Ac radioligand molecules can be observed, in some cases causing nephrotoxicity (**Figure 7B**) 36,50,95,96.

**Figure 8:**
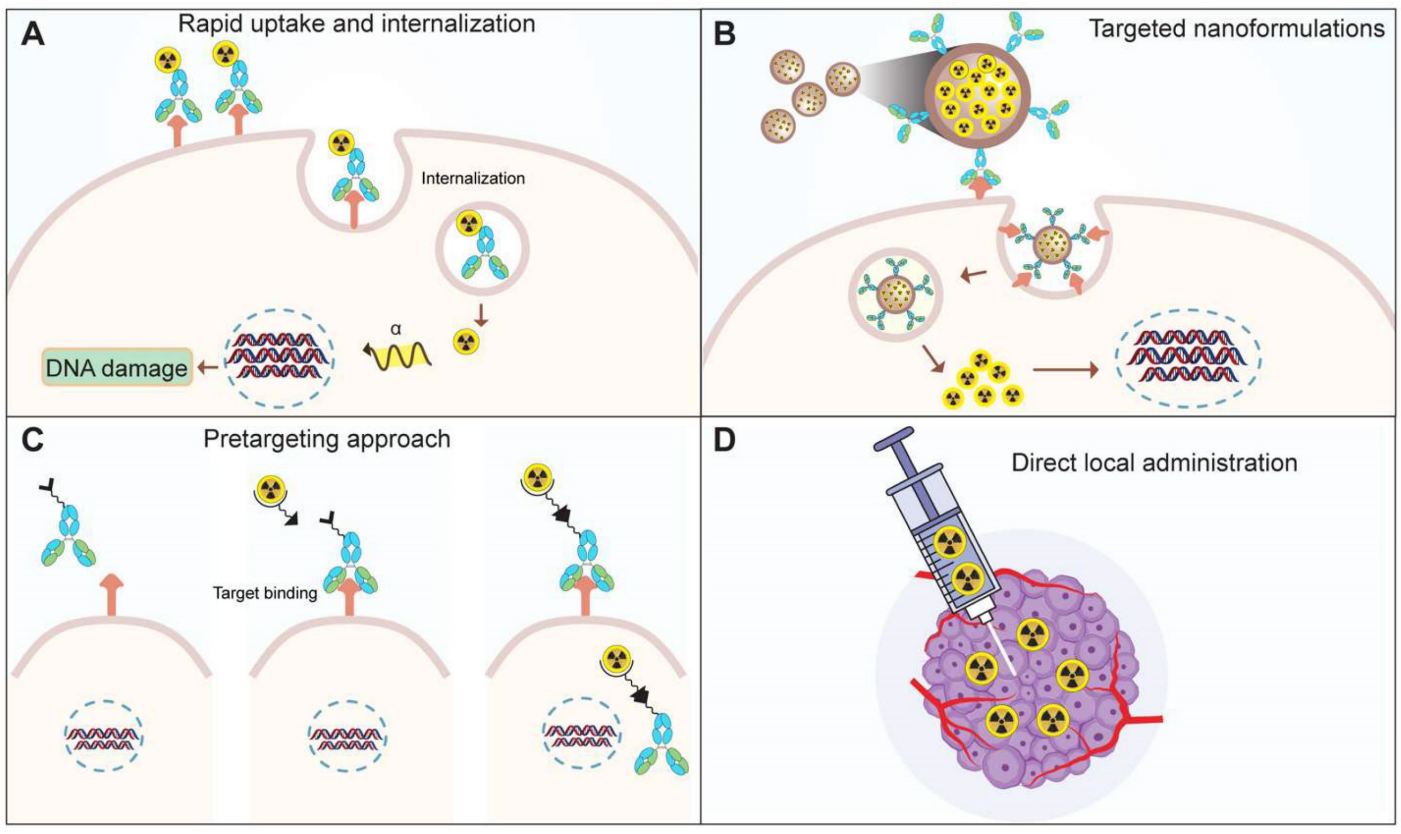
Strategies to mitigate the toxicity of recoiled daughters. **A**. Use of rapid uptake and internalizing targets, **B**. Encapsulation of the TAT radionuclide within nanoparticles, **C**. pretargeting, **D**. Intra-tumoral administration of radioactivity via injection.

To address the toxicity associated with recoiled daughters, researchers are investigating novel strategies (**Figure 8**). One approach involves facilitating the rapid uptake and internalization of alpha emitters within the target tissue (**Figure 8A**) 97. The use of high affinity and rapidly internalizing molecules may decrease the delivery of daughter isotopes to healthy organs 21,97,98. Secondly, the encapsulation of the nuclide within nanoparticles could minimize damage to normal cells (**Figure 8B**). Preclinical studies have explored the feasibility of loading radioisotopes in nanoformulations. Nanozeolites were successfully used as a carrier for ^224^Ra and ^225^Ra, along with their daughter radionuclides 99. Similarly, surface-modified, PSMA-targeted liposomes also demonstrated the usability of nanoformulations for the delivery of ^225^Ac to prostate cancer cells 100. In another approach, the layered nanoparticles consisting of ^225^Ac-doped {La0.5Gd0.5}PO4@GdPO4@Au were prepared to trap the daughters inside the core after decay 101. These gold-coated, layered nanoparticles, labeled with ^225^Ac, help retain ^213^Bi in the target tissue minimizing the kidneys uptake over time compared to the non-coated or non-targeted nanoparticles 101. Nano formulations allow site-specific and controlled delivery of the payload to target site. Thus, liposomes were utilized to deliver ^225^Ac and its radioactive daughters to ovarian cancer cells. The daughter isotopes were successfully retained in the liposome core, however the encapsulation efficiency was low 102. Similarly, folate-F(ab′)2 decorated liposomes allow targeted ^223^Ra delivery 103. In addition to targeting efficiency, the subcellular distribution of therapeutic molecules is crucial. A comparison was conducted to evaluate the cytotoxicity of PSMA and PSMA antibody-decorated liposomes loaded with ^225^Ac 104. The PSMA-decorated liposomes were three times more effective in inducing cell death, attributed to the delivery of the isotope to the perinuclear space. Ensuring stringent quality control measures is imperative in the development of nanocarriers for ^225^Ac delivery. Stability studies under relevant physiological conditions are essential to ascertain the long-term viability and performance of the nanocarriers. Addressing quality control and stability issues is paramount to guarantee the safety and efficacy of ^225^Ac-loaded nanocarriers, which could pave the way for their future translation into clinical applications 104-106.

Extensive research on radionuclide carriers, primarily antibodies, has predominantly focused on IgG-type antibodies. However, the commercial viability of radiolabeled antibodies faces challenges due to hematological and other toxicities associated with the prolonged circulation of antibodies 107. To overcome these challenges, alternative strategies including antibody fragments, recombinant proteins, and pre-targeting approaches have been explored. A noteworthy strategy is a pretargeting, where tumor cells are initially targeted with an unlabeled antibody 108. Subsequent administration of a radiolabeled compound binds to the antibody, facilitating targeted delivery (**Figure 8C**). Unbound radiolabeled molecules are swiftly excreted, contributing to a more precise and efficient therapeutic approach 109. Hapuarachchige et. al studied a pretargeting approach on PC3 cells where an anti-PSMA 5D3 mAb and its F(ab')2 fragments with trans-cyclooctene (TCO) were used as a pretargeting component 110. Subsequently, a drug delivery component, human serum albumin loaded with the DM1 anti-tubulin agent functionalized with PEGylated tetrazine (PEG4-Tz), was employed. The results demonstrated enhanced specific killing of PSMA(+) cells. Similarly, van Rij *et al.* employed the trivalent bispecific antibody TF12, composed of two anti-TROP-2 Fab fragments and one anti-histamine-succinyl-glycine (HSG) Fab fragment, for pretargeted theranostics. The delivery of the therapeutic agent, a radiolabeled hapten peptide ([^177^Lu]Lu-IMP288), demonstrated efficacy in treating TROP-2-expressing PC3 tumors 111. PET imaging using the same pair, [^68^Ga]Ga-IMP288 and TF12, enabled the detection of PCa tumors with improved contrast, showing lower bladder or kidney uptake compared to the commonly observed pattern with [^68^Ga]Ga-PSMA or FDG 111. Similar to this, a theranostic pair, using proteus-DOTA as a carrier for ^225^Ac and ^111^In was developed 112. This carrier was captured in vivo by a single-chain variable fragment (scFv) attached to the cell surface. This pretargeting study revealed high tumor accumulation and low uptake in normal tissues. The results were reproduced in solid human cancer xenograft models, including colorectal cancer, breast cancer, and neuroblastoma.

By injecting a radiolabeled molecule locally into the tumor tissue, redistribution of daughter isotopes can be minimized to some extent (**Figure 8D**) 21. The dose escalation study by Krolicki et. al for [^225^Ac]Ac-DOTA-SP have shown that the local injections of a radiopharmaceutical were safe for glioblastoma patient when administered at 10, 20 and 30 MBq per cycle for 6 cycles (2 month interval) 113. No hematological or renal toxicities were reported from this study 113. Similar to this, in a pilot study by Cordier *et al.* in five patients of gliomas were locally injected with [^213^Bi]Bi-DOTA-substance P. The [^213^Bi]Bi-DOTA-substance P treatment was found to be well-tolerated without toxicity 114. While direct administration is suitable for short half-life isotopes, caution is crucial for long half-life isotopes. The direct local administration route has been employed in the treatment of lung and pancreatic cancers in vivo 115,116. The prolonged presence of long-lived isotopes in the body increases the risk of cumulative radiation exposure to healthy tissues. Therefore, meticulous consideration and potentially modified administration strategies are essential to ensure the balance between therapeutic efficacy and minimizing adverse effects when dealing with long half-life isotopes.

### Radiobiological considerations, dose optimization, and fractionation

As the evidence supporting the utilization of ^225^Ac and other high-LET radioisotopes in cancer therapy accumulates, the consideration of the relative biological effectiveness (RBE) value becomes imperative 117. This significance is particularly highlighted when patients undergo one or more forms of radiation therapy, such as a combination of EBRT followed by TAT, or when patients resistant to [^177^Lu]Lu-PSMA-617 treatment are enrolled for [^225^Ac]Ac-PSMA-617 therapy. The high RBE values characteristic of high-LET isotopes stem from their induction of DNA double-strand breaks (DSBs), ultimately leading to cell death. Despite technological advancements allowing for escalated tumor doses, the current approach often involves prescribing the same dose uniformly to all patients, this could lead to underdosage or overdosage for specific patients. Consideration of the distribution and volume of metastases can help tailor TAT treatments to individual patients, optimizing efficacy and minimizing toxicity. Thus, studying the tumor cells before and after the radiation dose would help prescribe an individual radiation dosage. Moreover, the bystander effects of alpha therapy, which can trigger immune responses and non-targeted tumor-cell killing, may offer promising therapeutic benefits.

In the context of therapeutic dose vs toxicity, fractionation of a large therapeutic dose of ^225^Ac into smaller manageable doses could enhance safety. This fractionation strategy serves several purposes. Firstly, it helps reduce the potential for toxicity by allowing healthy tissues to recover between doses. Secondly, it provides a more precise and controlled approach to treatment, optimizing the therapeutic ratio, and maximizing the impact on cancer cells while minimizing harm to surrounding normal tissues. Thirdly, it aligns with radiobiological principles, taking advantage of the fact that cancer cells may be more susceptible to cumulative radiation damage over multiple exposures. The fractionated dose strategy for ^225^Ac has been utilized in prostate cancer and multiple myeloma with promising results 36,70,118,119. While it is widely acknowledged that fractionation of therapy offers advantages, it is important to consider potential disadvantages as well. Fractionation, although beneficial, can present challenges such as complex implementation strategies, treatment interruption, increased cost, and the potential for delayed tumor regression.

### ^225^Ac Imaging techniques

The four alpha particles from the decay chain of ^225^Ac imparts a high therapeutic efficacy; however, current studies indicate the hurdles of targeting affected tissue with the entire decay chain. Thus, it becomes imperative to confirm the presence of the daughter isotopes at the target tissue after the injections of ^225^Ac-based radiopharmaceuticals. In preclinical studies, the ex-vivo biodistribution analysis of ^213^Bi and ^221^Fr is difficult to complete due to their short half-lives. The gamma emissions of daughters, including the 440 keV (^213^Bi) and 218 keV (^221^Fr) peaks for SPECT imaging, remain valuable for dosimetry assessment and therapy response evaluation; however, the low therapeutic ^225^Ac activity administered, typically 100 kBq/kg, result in limited gamma emissions for imaging 120. Alternatively, much progress has been made in the small-scale organ dosimetry for tumor models used in preclinical studies, which can be extrapolated to human subjects 121. This involves ex-vivo autoradiography of alpha particle decay events, generating a real-time dose-rate map that provides information about the spatial distribution of the radioactivity in the tissue 121. Traditional collimator-based SPECT imaging has challenges to image gamma emission above 300 keV, therefore utilization of 440 keV from ^213^Bi is more difficult. To overcome this, a new approach to combine the coded aperture imager (for lower keV) and Compton imager (for higher keV) was utilized to visualize and quantify the daughters from ^225^Ac 122.

Quantitative single photon emission computed tomography (SPECT) of gamma emissions at 440 keV and 218 keV peaks allows image-based dosimetry to determine the absorbed dose. Although posttherapy imaging is challenging for alpha emitters, one study presented a case of mCRPC treated with [^225^Ac]Ac-PSMA-617. The posttherapy scans, utilizing 3 different energies 78, 218, and 440 keV showed successful targeted therapy, and distribution was visualized 123. Imaging with 3 major photopeaks (78, 218, and 440 keV) enhanced image quality compared to the previously reported 440 and 218 keV photopeaks 124. Recently, a quantitative SPECT imaging for [^225^Ac]Ac-PSMA-I&T therapy for mCRPC patients was demonstrated with image-based estimations of the absorbed dose 125. These prior and ongoing studies indicate promise for direct imaging of the daughters of ^225^Ac.

## Other Alpha Particle Emitting Isotopes for TAT in PCa

### Thorium-227

**Figure 9:**
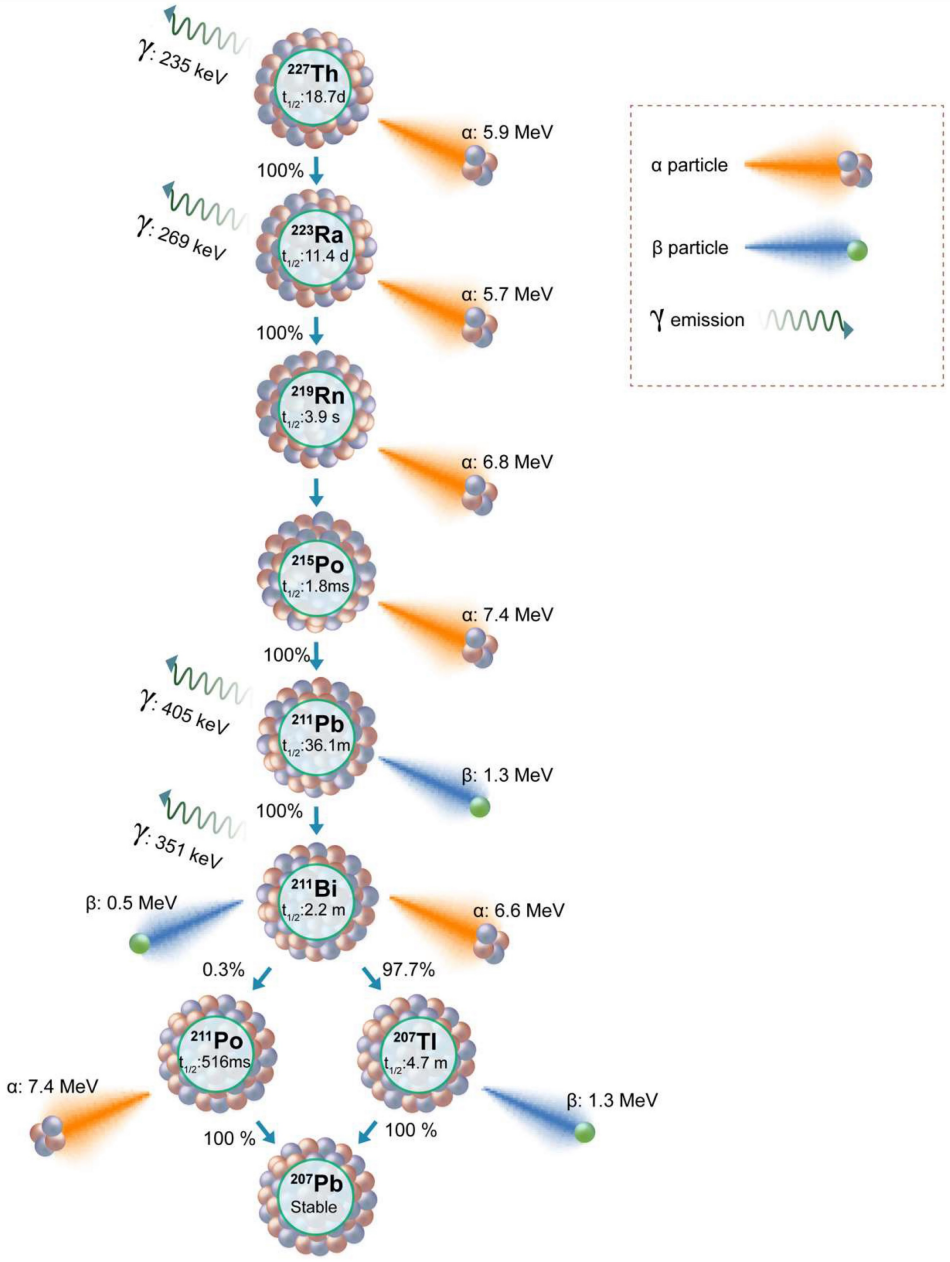
Schematic presentation of the decay series of Thorium-227 and Radium-223.

Targeted Thorium-227 conjugates (TTCs) represent another class of therapeutic radiopharmaceuticals meticulously designed for precision in TAT. The TTC process involves binding the alpha-emitting Thorium-227 (^227^Th) to a chelator, strategically linked to a monoclonal antibody (MAb) or small molecules engineered for tumor targeting 126. ^227^Th, belonging to the actinide series (half-life of 18.7 days), follows a decay scheme featuring a cascade of alpha (five alpha particles) and beta emissions, culminating in the formation of the stable isotope ^207^Pb (**Figure 9**) 127. The alpha particle emitted during the decay of ^227^Th possesses a mean energy of 5.9 MeV, contributing to a total energy of 34 MeV from all the five alpha particles in the decay series. Additionally, ^227^Th serves as the precursor to Radium-223 (Xofigo), an alpha-emitting radionuclide globally approved for clinical use in CRPC. In contrast to ^223^Ra, the precursor radionuclide ^227^Th demonstrates the ability to form stable chelator complexes, making it well-suited for targeted radioimmunotherapy. The extended half-life of ^227^Th not only streamlines practical handling and administration but also proves advantageous for achieving elevated radiation doses at the tumor site. This prolonged half-life contributes to enhanced efficiency in the production process and allows for optimized scheduling in therapeutic applications.

The radiolabeling of ^227^Th has posed a substantial hurdle in the progress of TTC. Initial investigations utilizing DOTA necessitated elevated temperatures (60^o^C) for efficient radiolabeling, a condition poorly suited for macromolecules like antibodies 128,129. This underscores the demand for innovative methodologies to improve radiolabeling efficiency, particularly when dealing with larger biological entities. To overcome this challenge, Ramdahl *et al.* introduced a solution by employing HOPO (2,3-hydroxypyridinone) for the chelation of ^227^Th at room temperature, offering a promising avenue for addressing the issues in the context of TTC development 130. Recently, Hammer *et al.* reported the use of PSMA-TTC for the treatment of preclinical models of PCa 131. PSMA-TTC was synthesized by conjugating the HOPO chelator to PSMA targeting full-length antibody (BAY 2315158) for radiolabeling. This PSMA-TTC exhibited selective binding and internalization in the cells expressing PSMA, resulting in DNA damage and apoptosis from alpha particle-mediated toxicity. PSMA TTC was also effective for the treatment of AR-sensitive (LNCaP and ST1273) and AR-resistant (KUCaP-1) in-vivo tumor models. The conjugate was specifically effective in inhibiting cancer progression and abnormal bone growth in a bone metastasis model 131.

Given that PCa predominantly depends on androgens for growth, concurrently addressing androgen receptor signaling alongside PSMA-TTC treatment emerges as a promising approach for prostate cancer therapy. Hammer *et al.* expanded their investigations to assess the synergies between PSMA-TTC and darolutamide, an androgen receptor inhibitor 132. The combined therapy involving PSMA-TTC and darolutamide exhibited synergistic effectiveness in the *in-vitro* as well as *in-vivo* tumor models. AR inhibition from darolutamide increased the PSMA expression thereby increasing the tumor-targeted delivery of PSMA-TTC and the therapeutic response 132. Currently, the clinical trial to test the safety, tolerability, pharmacokinetics, and antitumor action of a PSMA-TTC is ongoing (NCT03724747). In addition to this, a combination of the PARP inhibitor (Olaparib) and PSMA-TTC is also being tested with synergistic anti-tumor results in various tumor models 133. In addition to this, development of novel peptidomimetic small molecules is also being designed to target PSMA for the delivery of ^227^Th. A recent study by Böhnke *et al.* involved the synthesis of mono- and multimeric (di-, tri-, and tetramers) PSMA-targeting small molecules with modified carboxylated HOPO for the treatment of PCa 134. The synthesized carboxy-HOPO exhibits heightened hydrophobicity and demonstrates robust labeling efficacy at room temperature. Among the studied forms of PSMA TTC, both monomeric and dimeric configurations exhibited exceptional tumor-to-background ratios with minimal accumulation in the kidneys. In contrast, the larger size of the trimer and tetramer led to increased accumulation in the liver 134. Despite challenges in radiolabeling, innovative approaches like HOPO chelation at room temperature and synergistic treatments with androgen receptor inhibitors offer exciting avenues for further development and clinical exploration.

### Radium-223

The ALSYMPCA trial played a pivotal role in establishing the therapeutic merit of Radium-223 (Xofigo), leading to its regulatory approval and emergence as a groundbreaking intervention for individuals with bone metastases. Radium-223's remarkable ability to selectively target bone metastases has led to improved overall survival rates and a significant delay in the development of symptomatic skeletal lesions 135. As illustrated in **Figure 9** decay scheme, ^227^Th undergoes alpha particle emission to transform into ^223^Ra, initiating a shared decay pathway between ^227^Th and ^223^Ra with the remaining daughter isotopes. ^223^Ra has a half-life of 11.4 days, resulting in a net of four alpha particles and two beta particles. Injection of the ^223^Ra and ^89^Sr resulted in increased bone uptake in the osseous site, without affecting the normal bone marrow suggesting its bone-seeking property 136. Subsequent preclinical investigations consistently validated the bone-targeting efficacy of ^223^Ra chloride in metastatic tumors 137. The clinical trials focused on toxicity and dose estimations and therapeutic effects of administration of Radium-223 to prostate cancer patients proved the efficacy for use in humans 138-140. The unique bone-targeting properties of ^223^Ra, demonstrated through preclinical investigations and clinical trials, indicate its efficacy and safety in treating prostate cancer patients.

### Astiatine-211

**Figure 10:**
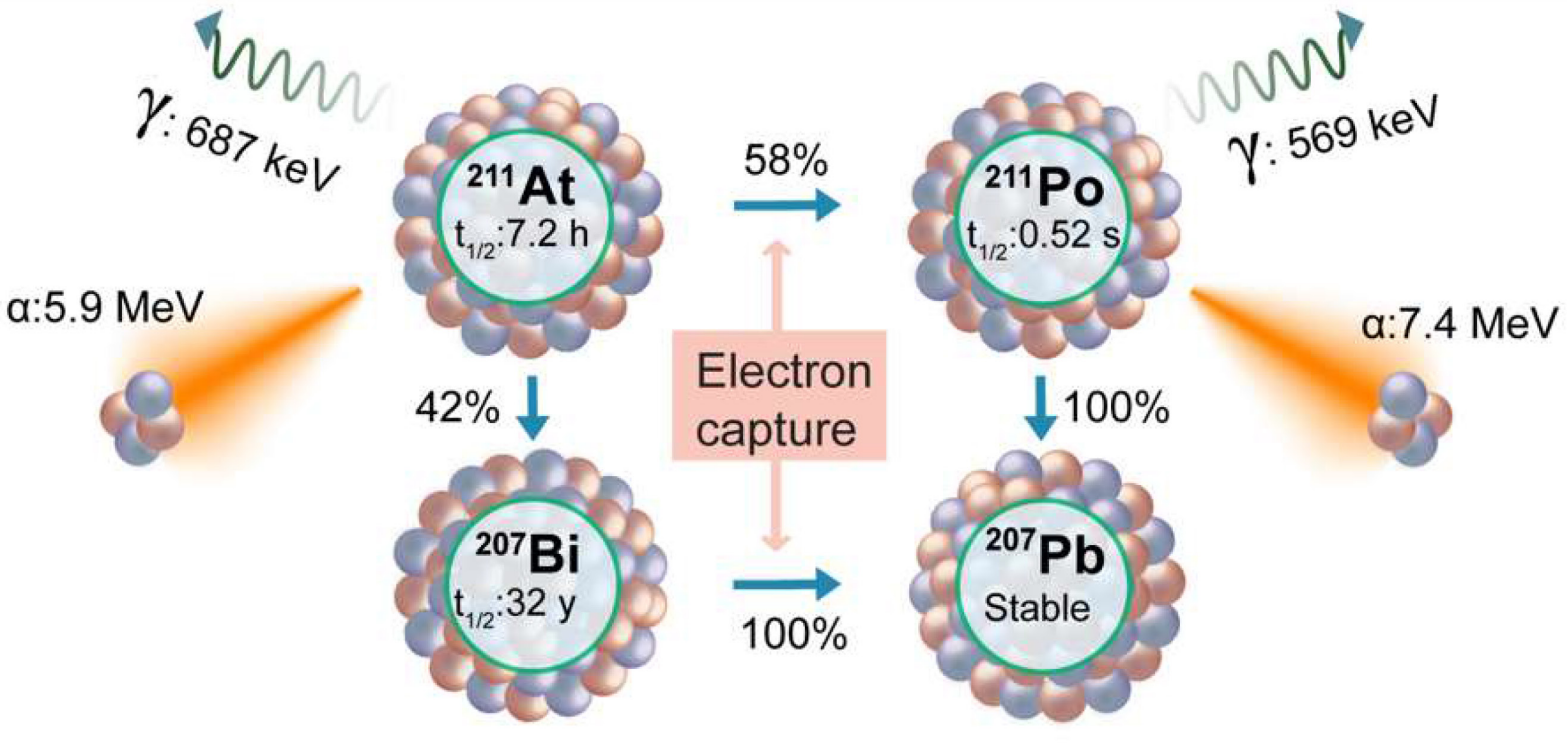
Decay scheme of Astatine-211.

Astatine-211 (^211^At) stands out as a compelling TAT radioisotope, presenting a judicious balance of favorable characteristics 141. With a half-life of 7.2 hours, ^211^At is endowed with an optimal temporal window for therapeutic applications. Importantly, unlike the other TAT isotopes, its decay series exhibit an absence of toxic daughters, a crucial consideration in ensuring the safety and efficacy of cancer treatments. Furthermore, ^211^At offers the practical advantage of feasible production in clinically relevant amounts, enhancing its accessibility for potential clinical applications. These attributes collectively position ^211^At as a promising contender in the expanding range of alpha-emitting radionuclides for targeted therapies. The decay process of ^211^At involves two branches (**Figure 10**). The first decay branch (42% decay) leads to the generation of ^207^Bi (t½ = 32.9 years) and the emission of an α-particle (5.9 MeV). The ^207^Bi subsequently decays by electron capture to ^207^Pb. The second decay branch (58% decay) facilitated by electron capture produces ^211^Po (t½ = 516 milliseconds), which subsequently undergoes decay to ^207^Pb, emitting an α-particle (7.4 MeV).

There are a few ongoing and planned clinical trials with ^211^At for other malignancies; however, the development of a TAT agent with ^211^At for PCa treatment is still in the preclinical stage. Early reports of development of PSMA-targeted (2S)-2-(3-(1-carboxy-5-(4-^211^At-astatobenzamido) pentyl)ureido)-pentanedioic acid ([^211^At]At-**6**) show that [^211^At]At-**6** exhibited significant tumor growth delay and improved survival in a PSMA-positive xenograft as well as a micrometastatic model 142. Similarly, Vaidyanathan *et al.* also studied Glu-urea based PSMA ligands containing a trialkyl stannyl group labeled with ^211^At by electrophilic astatodestannylation 143. These urea-based molecules showed tumor accumulation with various degrees of dehalogenation resulting in thyroid, kidney, and stomach accumulation 142,143. The radiolabeling method utilized in these studies results in lower molar activity. To address this challenge, a new methodology has been presented by Shirakami *et al.* for the synthesis of PSMA-targeted ligands 144. This innovative approach entails a substitution reaction wherein dihydroxyboryl groups are replaced with ^211^At on PSMA ligands. Although the synthesized molecules ([^211^At]At-PSMA1, [^211^At]At-PSMA5, and [^211^At]At-PSMA6) show high tumor retention; due to dehalogenation, as well as due to PSMA expression at proximal tubules, significant toxicity was seen in healthy organs including kidneys 145,146. In addition to this, peptide formulation labeled with the ^211^At has been tested to achieve TAT by targeting bombesin receptor 147. The bombesin targeting [^211^At]At-AB-3 molecules failed to accumulate in the tumor to achieve sufficient concentration, possibly due to stability and dehalogenation issues.

To address potential PSMA negativity and heterogeneity, Back *et al.* tested the anti-PSCA A11 minibody to achieve targeted delivery of the ^211^At to prostate stem cell antigen (PSCA) 148. PSCA is overexpressed in localized as well as metastatic prostate cancer. [^211^At]At-A11 showed significant therapeutic action for both subcutaneous as well as tibia tumor models. Interestingly, the blocking of sodium iodine importer with sodium perchlorate significantly reduced the uptake of free ^211^At in healthy organs, minimizing the off-target toxicity. In summary, ^211^At shows promise for Targeted TAT in PCa with favorable features, optimal timing, and no toxic byproducts.

### Bismuth-213

The integration of Bismuth-213 (^213^Bi, half-life= 45.6 minutes) into TAT gained significant traction through the pioneering efforts of the Memorial Sloan Kettering Cancer Center in developing a ^225^Ac/^213^Bi generator. This generator, a pivotal advancement, enabled on-site production of ^213^Bi, thereby improving accessibility for its application in the treatment of patients with leukemia 149,150. Early investigations using ^213^Bi-labeled J591 ([^213^Bi]Bi-J591) demonstrated encouraging outcomes in terms of targeted binding and effective tumor cell eradication in in-vitro cells, spheroids, and in-vivo tumor models 151-153. These findings affirmed the viability of ^213^Bi in TAT, particularly for metastatic microtumors, where high doses of radiation per cell are crucial for achieving complete eradication of tumor cells, additionally, highlighting that larger solid tumors show response but the complete regression may not be achieved 154,155. In addition to PSMA targeting with J591, another potential target investigated for the delivery of ^213^Bi is the plasminogen activator inhibitor type 2 (PAI2) protein, known to be overexpressed on prostate cancer cells. The [^213^Bi]Bi-PAI2 conjugate demonstrated induced cell death through mechanisms involving DNA damage and apoptosis, while concurrently exhibiting low or negligible toxicity 154. Yong Li and colleagues proposed the concept of multiple targeted radioimmunotherapy conjugates, wherein multiple proteins were targeted using ^213^Bi radiolabeled vectors for respective proteins 155-157. The cocktails of ^213^Bi-labeled monoclonal antibodies against C595, BLCA-38, and J591, along with [^213^Bi]Bi-PAI2, were tested on PCa cells, suggesting that the cocktails exhibited superior efficacy compared to monotherapies. However, for the personalized application of these combinations, information on target expression is imperative to determine the combination that would work effectively 155,156,158.

In addition to the high molecular weight macromolecules, utilizing small molecular weight vectors like PSMA or nanobodies for ^213^Bi delivery offers distinct advantages 159. These vectors, due to their compact size, enhance tissue penetration, exhibit rapid clearance from non-target sites, and efficiently distribute within tumors. Additionally, the biological half-life of the vector aligns with that of ^213^Bi, resulting in rapid blood clearance reducing radiation exposure, and lowering the risk of hematologic toxicity 160. In this context, the first-in-human study with [^213^Bi]Bi-PSMA-617 showed a remarkable response (PSA decreased to 43 µg/mL from 237 µg/L) when treated with two cycles of a combined 592 MBq dose 161,162. Additionally, patients resistant to [^177^Lu]Lu-PSMA-617 exhibited a substantial anti-tumor response with minimal hematological toxicity when treated with [^213^Bi]Bi-DOTATOC 163. An additional advantage of ^213^Bi, in comparison to ^225^Ac, is its absence of daughter isotopes, eliminating concerns related to recoiling. This characteristic simplifies the dosimetry calculations and ensures a more controlled and predictable radiation delivery in targeted alpha particle therapy. A key challenge in advancing ^213^Bi-based TAT lies in the availability of a reliable ^225^Ac/^213^Bi generator.

The successful establishment and operation of a reliable ^225^Ac/^213^Bi generator are indispensable for securing a sustainable and accessible supply of ^213^Bi, thereby facilitating the ongoing advancement and widespread adoption of this promising therapeutic modality 18,164. The growing number of sites dedicated to accelerator-produced ^225^Ac holds promise in addressing and resolving this challenge, offering a potential solution to ensure the availability and accessibility of ^213^Bi for medical applications.

### Lead-212

An additional noteworthy in situ α-emitting radiometal is Lead-212 (^212^Pb, 10.6 days half-life). Its decay involves α and β- particle emission to produce stable ^208^Pb. Utilizing ^212^Pb offers the advantage of employing the ^203^Pb as an imaging surrogate, facilitating imaging and dosimetry estimations. In clinical settings, ^212^Pb labeled trastuzumab has been tested in ovarian cancer patients with peritoneal carcinomatosis, and it demonstrated notable antitumor activity with good tolerability 165,166. Similarly, in a preclinical study, a single intravenous dose of [^212^Pb]Pb-trastuzumab resulted in a substantial reduction in tumor growth with prolonged survival in mice with PC3 tumors, importantly, without loss of body weight 167. The first human study with PSMA targeted ^212^Pb involved the use of urea-based ligands with p-SCN-Bn-TCMC or DO3AM chelators 168. Two patients with mCRPC were injected with ^203^Pb labeled CA012, and dosimetry estimates were acquired, revealing an estimated effective dose of 6-7 mSv from 250-300 MBq of [^203^Pb]Pb-CA012. In-vivo studies have also explored the use of ^212^Pb for labeling PSMA-directed small molecules or biomarker-specific antibodies for the treatment of preclinical prostate cancer tumors 159.

### Terbium-149

Terbium-149 (^149^Tb) is an isotope with favorable properties for theranostics. ^149^Tb has relatively short half-life (t½ = 4.1 hours) and decays by electron capture (76%), alpha (17%), and positron (7%) emissions. The alpha particles from ^149^Tb possess 25-28 μm path length, with 140-142 keV/μm LET 169. The shorter half-life of ^149^Tb is considered optimal for delivery with PSMA ligands with faster excretion 169. Utilizing the positrons emissions from ^149^Tb, Müller and coworkers developed “alpha-PET” with ^149^Tb labeled PSMA-617 ([^149^Tb]Tb-PSMA-617) for treatment and imaging of PSMA expressing PC3-PIP xenografts 170. [^149^Tb]Tb-PSMA-617 was selectively internalized in PC3-PIP tumor xenografts and was successfully imaged with PET/CT scans. A single dose (6 MBq) or fractionated (2 x 3MBq) treatment of [^149^Tb]Tb-PSMA-617 resulted in delayed tumor growth, and improved survival compared to untreated control 170. Considering the lower half life and faster excretion, multiple cycles of ^149^Tb labeled agents would be beneficial, however; this would require in house production. In addition to the supply, another concern for ^149^Tb is about the long-lived daughter isotopes. The daughter isotopes ^149^Gd (t½ =9.3 days), ^145^Sm (t½ = 340 days) ^145^Eu (t½ =93 days) from ^149^Tb decay require more long term safety studies 171. Various preclinical studies have shown the use of ^149^Tb alpha therapy for treatment of pancreatic 169, breast 172, and hematological malignancies 173; suggesting great potential for ^149^Tb-based radiopharmaceutical research.

## Conclusion

Recent and notable clinical outcomes in the treatment of advanced-stage cancers with beta- and alpha-emitting radiopharmaceuticals have sparked increased interest in radiopharmaceutical therapy. The approval of [^177^Lu]Lu-PSMA-617, coupled with emerging evidence in receptor-targeted therapy that capitalizes on the unique properties of cancer cells, underscores the potential for developing novel therapeutic strategies. Actinium-225, known for high LET, longer half-life, and shorter path length has emerged as an important alpha emitter for various malignancies, including prostate cancer. Therefore, [^225^Ac]Ac-PSMA-617 therapy results have been considered as one of the promising options for treatment- or [^177^Lu]Lu-PSMA-617- resistant patients. To enhance the therapeutic efficacy of PSMA-based radionuclide therapy, diverse strategies are being explored in clinical settings. These strategies encompass developing refined patient selection methods, escalated radiation damage through dosimetry-guided dose selection, or utilization of α-emitters in place of β-emitters. Additionally, combined approaches aimed at overcoming radioresistance mechanisms, whether intrinsic or acquired, are being investigated. These multidimensional strategies involve novel hormonal agents, PARP inhibitors, and immunotherapy, all geared toward enhancing the effectiveness of PSMA-based radionuclide therapy. However, challenges persist as a vast number of mCRPC patients show no response to [^177^Lu]Lu-PSMA-617 RLT. Heterogeneity in PSMA expression, mechanisms of resistance, and the potential impact of PSMA-targeted therapies on non-prostate tissues necessitate further investigation. While [^177^Lu]Lu-PSMA-617 and [^225^Ac]Ac-PSMA-617 treatments have enhanced the overall prognosis for patients, the emergence of resistance within tumors has remained an issue, leading to ongoing disease progression. The length of responses to RLT is frequently brief, even among patients who are initially responsive, and the underlying reasons for resistance remain unknown. Inadequate radiation dose delivery has been established as a factor contributing to resistance, particularly for RLT with β-emitters. Diffuse marrow infiltration is a typical post-treatment progression pattern with [^177^Lu]Lu-PSMA-617, which could be caused by insufficient radiation dose distribution to small-volume illness. Given that [^177^Lu]Lu-PSMA-617 treatment failure is frequently related to the advancement of micrometastatic illness, the use of other radionuclides with shorter route lengths may be a viable approach. Single traversals of alpha particles or Auger electrons can trigger cytotoxic double-stranded DNA breaks in the nucleus, thereby bypassing the constraints associated with micrometastatic disease resistance shown with [^177^Lu]Lu-PSMA-617.

The successful development of radiopharmaceuticals relies on efficient chelators and tumor-specific targeting vectors. Chelators tested for radiolabeling of ^225^Ac are EDTA, PEPA, TETPA, DTPA, DOTA, and Macropa, among others. Among these, the DOTA and Macropa are the most favored to produce higher radiolabeling yield with suitable stability. The review brought attention to the significance of biomarkers in prostate cancer, with a particular focus on the commonly used biomarker PSMA. Despite its conventional use, ongoing studies to develop alternative PSMA ligands, peptides, or albumin modifications underscore the continuous exploration and refinement of diagnostic approaches related to PSMA. As research advances, the identification and utilization of biomarkers will likely play a pivotal role in enhancing diagnostic accuracy and tailoring more effective therapeutic interventions. In this regard, various new biomarkers are being tested for PSMA-negative prostate cancer, most notable novel biomarkers being tested are CD46, human KLK3, and VE-cadherin. DLL3, CDCP1 or GRPR are a few other promising targets that can be used for alpha therapy in PSMA-negative tumors.

The review highlights challenges in ^225^Ac-based therapies, including isotope production, recoiling energy, daughter redistribution, and the need for improved imaging techniques. These hurdles represent crucial areas for further research and development to unlock the full potential of ^225^Ac-based therapies. To improve the efficacy of current TAT agents, various combination approaches were discussed including combinations with PARP inhibitors, anti-androgens, and immune modulators. We also shed light on alternative isotopes, such as ^227^Th,^ 223^Ra, ^211^At, ^213^Bi, ^212^Pb, and ^149^Tb suggesting a diversified landscape for potential alpha-emitting radionuclides in TAT for prostate cancer. These alternatives provide avenues for further research, offering potential solutions to some of the obstacles associated with ^225^Ac. While the application of actinium-225 in prostate cancer therapy holds tremendous potential, further research and development are vital to address the current limitations and optimize its use. The evolving landscape of alpha-particle therapy, with ^225^Ac at its forefront, signifies a paradigm shift in the way we approach and treat prostate cancer. As research continues to advance, the prospect of realizing the full therapeutic potential of actinium-225 in clinical settings becomes an exciting and achievable goal.

## Figures and Tables

**Table 1:** Radiolabeling conditions and radiochemical yields for chelators used for ^225^Ac complexes.

Chelators	Buffers	pH	Temperature	Radiochemical Yield	Reference
EDTA, PEPA, CHX-PEPA	1M NH_4_OH	5	40°C for 30 min	80-90%	22
HEHA	0.15M NH_4_OH	4 to 7	37°C for 30 min	60-85%	24
DTPA, TETA, DOTPA	3 M NH_4_Ac	6	37°C for 0, 30, 60, and 120 min	Very low	25
DOTA and DOTMP	3M NH_4_Ac	6	0, 30, 60, and 120 min	100% and 78%	25
Macropa	0.15M NH_4_Ac	5.5 to 6	37°C for 5 min	96%	26

**Table 2:** Current or active clinical trials with ^225^Ac based monotherapy or combination therapy.

Monotherapy					
**Therapy**	**Trial number**	**Patients**	**Previous therapy**	**Parameters**	**References**
^[225^Ac]Ac-J591	NCT03276572	mCRPC	anti-Androgen therapy	Dose escalation	69
[^225^Ac]Ac-J591	NCT04506567	mCRPC	anti-Androgen therapy	Fractionation, MTD	70
[^225^Ac]Ac-PSMA-I&T	NCT05219500	mCRPC	anti-Androgen therapy	Safety and Efficacy	73
[^225^Ac]Ac-PSMA-617	NCT04597411	PCa	Naïve and Pretreatment with 177Lu-PSMA-617	Dose escalation	74
[^225^Ac]Ac-PSMA-617	NCT05567770	Hormone-sensitive metastatic PCA	Naïve and prior curative-intent treatment to the prostate	Dose-limiting toxicities, MTD	75
**Combinations**					
[^225^Ac]Ac-J591 + ^177^Lu-PSMA I&T	NCT04886986	mCRPC	Anti-androgen therapy, Chemotherapy	Dose-limiting toxicity, dose escalation	72
[^225^Ac]Ac-J591 + Pembrolizumab	NCT04946370	mCRPC	anti-Androgen therapy	Safety, dose-limiting toxicity	76
